# Mapping the co-localization of the circadian proteins PER2 and BMAL1 with enkephalin and substance P throughout the rodent forebrain

**DOI:** 10.1371/journal.pone.0176279

**Published:** 2017-04-19

**Authors:** Ariana Frederick, Jory Goldsmith, Nuria de Zavalia, Shimon Amir

**Affiliations:** 1 Centre for Studies in Behavioural Neurobiology, Concordia University, Montreal, Quebec, Canada; 2 Department of Biology, Concordia University, Montreal, Quebec, Canada; 3 Department of Psychology, Concordia University, Montreal, Quebec, Canada; Pennsylvania State University, UNITED STATES

## Abstract

Despite rhythmic expression of clock genes being found throughout the central nervous system, very little is known about their function outside of the suprachiasmatic nucleus. Determining the pattern of clock gene expression across neuronal subpopulations is a key step in understanding their regulation and how they may influence the functions of various brain structures. Using immunofluorescence and confocal microscopy, we quantified the co-expression of the clock proteins BMAL1 and PER2 with two neuropeptides, Substance P (SubP) and Enkephalin (Enk), expressed in distinct neuronal populations throughout the forebrain. Regions examined included the limbic forebrain (dorsal striatum, nucleus accumbens, amygdala, stria terminalis), thalamus medial habenula of the thalamus, paraventricular nucleus and arcuate nucleus of the hypothalamus and the olfactory bulb. In most regions examined, BMAL1 was homogeneously expressed in nearly all neurons (~90%), and PER2 was expressed in a slightly lower proportion of cells. There was no specific correlation to SubP- or Enk- expressing subpopulations. The olfactory bulb was unique in that PER2 and BMAL1 were expressed in a much smaller percentage of cells, and Enk was rarely found in the same cells that expressed the clock proteins (SubP was undetectable). These results indicate that clock genes are not unique to specific cell types, and further studies will be required to determine the factors that contribute to the regulation of clock gene expression throughout the brain.

## Introduction

The generation of behavioral and physiological circadian rhythms in animals is governed by clock genes that mediate cell-autonomous and tissue-level circadian oscillations in gene expression. In mammals, circadian clock genes are found in most tissues throughout the body, including the brain. The interaction between clock genes and neural function is important for understanding normal behaviour, as well as disease development [1–3]. The suprachiasmatic nucleus (SCN), located in the ventral hypothalamus, plays a central role in coordinating rhythms throughout the body and synchronizing them to the environmental light-dark cycle [3–5]. Clock gene rhythms have been reported in many other brain regions [6–10] and are thought to play a complementary role in fine-tuning the timing of daily behaviours [3, 11]. In contrast to the well-studied SCN, very little is known about the mechanisms that regulate clock gene expression in these downstream regions. One important step in addressing these questions is to determine the extent to which clock genes are expressed across a variety of neural cell types.

Recent evidence points to daily fluctuations in hormone levels and neurotransmitter secretion as key entrainment signals outside of the SCN [12–14]. It has been demonstrated that perturbations of specific neurotransmitters or hormones can disrupt clock gene function in discrete brain areas. For example, blocking glucocorticoid or thyroid hormone signaling blunts peak expression of the core clock protein PER2 in the oval nucleus of the bed nucleus of the stria terminalis (BNSTov) and the central nucleus of the amygdala (CEA) [15–17]. In adrenalectomized animals, replacement of glucocorticoid hormone through timed daily injections or by adding corticosterone to the drinking water is able to re-entrain PER2 rhythms independently of the SCN [18, 19]. Similarly, dopamine depletion via 6-hydroxydopamine lesion impairs PER1 and PER2 rhythms in the dorsal striatum and BNSTov [20, 21]. This effect has been linked to D2-receptor signalling, as a similar blunting of the PER2 rhythm can be induced by constant infusion of a D2-receptor antagonist, and timed daily injection of a D2-receptor agonist, though not a D1 receptor agonist, restores and entrains a PER2 rhythm in the dopamine-depleted striatum [20].

These findings suggest that clock gene expression is responsive to specific neurotransmitters or hormones. This has lead us to question whether extra-SCN clock gene expression might be localized to distinct neuronal subpopulations throughout the forebrain. In order to study the distribution of clock genes in specific cell types within the CNS, we chose to anatomically determine the co-expression of PER2 and BMAL1 with two neuropeptides that have differing expression patterns throughout the rodent forebrain. For comparison, we chose the neuropeptides substance P (SubP) and Enkephalin (Enk), since they represent specific cell-types in our brain regions of interest. Our initial analysis focused on the limbic forebrain where, in the dorsal striatum and nucleus accumbens, SubP and Enk are markers for D1- and D2-receptor bearing medium spiny neurons, respectively [22, 23]. Similarly, the two main neuronal populations of the CEA and BNSTov produce either Enk or corticotropin releasing hormone (CRH) [24]. Our findings in these limbic areas suggested that clock genes were found in nearly all neurons and were not restricted to either cell type. The same tissue was then further analyzed to address if a similar pattern was found in other brain regions. The parvocellular region of the paraventricular nucleus of the hypothalamus (PVH) expressed low levels of SubP and moderate levels of Enk. In this region Enk partially overlaps with expression of CRH, thyrotropin releasing hormone (TRH) and oxytocin [25–27]. Similarly, the arcuate nucleus (Arc) expresses low levels of SubP and moderate levels of Enk, both of which are known to be a limited to a specific subset of neurons [28–31]. In the thalamus, the dorsal medial portion of the medial habenula (HabM) does not express Enk, but has a large population of cholinergic neurons that also express SubP [32]. No other thalamic or hypothalamic regions were identified to strongly express BMAL1 and PER2 with either peptide of interest. Apart from the olfactory bulb (OB), we found that the trend continued throughout these regions and most neurons expressed clock genes.

## Methods

### Animals

All experimental procedures were conducted under the guidelines of the Canadian Council of Animal Care and approved by the animal care ethics committee at Concordia University, Quebec. Concordia University Animal Research Ethics Committee approval number: 30000256. Eight male Wistar rats (Charles River, St-Constant, QC), weighing 300–350 g, were used for this project. Prior to the start of the experiment, animals were housed individually under a 12h light/dark cycle for two weeks in lightproof boxes with *ad libitum* access to food and water. They also had free access to running wheels, monitored continuously using VitalView software (Mini-Mitter, Sunriver, OR). The experiment began after all animals’ activity entrained to the light/dark cycle.

### Colchicine

To enhance cell body visualization, colchicine was injected into the left lateral ventricle 24–36 h prior to perfusions [33]. Animals were anesthetized with a mix of ketamine (Ketaset; 90mg/kg, i.p.; Ayerst) and xylazine (Rompun, 10 mg/kg, i.p.; Bayer), and then 100 μg of colchicine (Sigma-Aldrich, Oakville, ON) dissolved into 5 μl of saline was infused at a rate of 0.5 μl/min over 10 min at the following stereotaxic coordinates: AP -3.0, ML 1.2, DV 3.6 (from the skull surface) [34].

### Tissue preparation and immunofluorescence

In order to image each brain area around the time of its peak PER2 expression, animals were perfused either one hour (ZT1) or ten hours (ZT10) after lights turned on (Table 1). Rats were deeply anesthetized with sodium pentobarbital (100 mg/kg, i.p.) and transcardially perfused with 300 ml of cold saline (0.9% NaCl), followed by 300 ml of cold paraformaldehyde (4% in a 0.1 M phosphate buffer, pH 7.3). Brains were extracted and stored overnight in paraformaldehyde at 4°C. Four serial sets of coronal sections were collected, sliced at a thickness of 30 μm using a vibratome and then stored at -20°C in Watson’s cryoprotectant [35] until ready to be used.

**Table 1:** Summary of tissue sampled for analysis.

Brain Area	n (PER2-Enk)	n (PER2-SubP)	n (BMAL1-Enk)	n (BMAL1-SubP)	Time of perfusion
Dorsal striatum, medial and lateral	3	3	3	3	ZT1
Nucleus accumbens, core and shell	4	3	3	3	ZT1
Olfactory tubercle	4	3	3	3	ZT1
Amygdala, central nucleus	3	3	3	3	ZT10
Amygdala, basolateral nucleus	-	-	-	-	ZT1
Bed nucleus of the stria terminalis, oval nucleus	3	4	3	3	ZT10
Bed nucleus of the stria terminalis, principal nucleus	-	3	-	3	ZT10
Hippocampus	-	-	-	-	ZT1
Suprachiasmatic nucleus	-	-	-	-	ZT10
Hypothalamic paraventricular nucleus	3	3	3	3	ZT10
Arcuate nucleus	3	-	3	3	ZT10
Medial habenula	-	3	-	3	ZT10
Olfactory bulb	3	-	3	-	ZT10

Each brain region was imaged at either one hour (ZT1) or ten hours (ZT10) following lights on, depending on when its maximum PER2 expression was expected. n = number of animals used in analysis

Double labeling was performed using the following four combinations: 1) PER2 with Enk, 2) PER2 with SubP, 3) BMAL1 with Enk and 4) BMAL1 with SubP. Only enough tissue that could be imaged within the next 5 days was processed at one time and therefore anterior and posterior sections were run at different times (stopping just posterior to the SCN, around -1.80 mm from bregma). In each run, free-floating sections were rinsed once for 10 min in phosphate buffered saline (PBS, pH 7.4), followed by 3x10 min rinses in 0.3% Triton-X in PBS (PBS-TX). Tissue was pre-blocked for 1 h at room temperature with gentle agitation in a solution of PBS-TX with 3% skim milk powder and 6% normal donkey serum (NDS) then directly transferred to the primary incubation. Tissue was incubated for 48 h with the primary antibody at 4°C with gentle agitation, rinsed 3x10 min in PBS-TX, then incubated with the secondary antibody for 1 h at room temperature with gentle agitation. Antibodies were diluted in a solution of 0.3% PBS-TX with 3% skim milk powder and 2% NDS. The following antibodies and dilutions were used: PER2 rabbit polyclonal 1:800 (Alpha Diagnostics, PER21-A, San Antonio, Tx), BMAL1 rabbit polyclonal 1:800 (Novus Biologicals, NB100-2288 Lot A-2, Littleton CO), Leu /Met-enkephalin [NOC1] mouse monoclonal 1:800 (Abcam, Toronto, ON), Substance P [SP-DE4-21] mouse monoclonal 1:400 (Abcam, Toronto, ON), anti-rabbit secondary Alexa-488 and anti-mouse secondary Alexa-594 1:500 (Life Technologies, Carlsbad CA). All antibodies used have been validated elsewhere [20, 36].

Once all incubations were complete, the tissue was rinsed 3x10 min in PBS-TX, then treated to reduce autofluorescence with Sudan Black B (Sigma-Aldrich, Oakville, ON): 0.1% in 70% ethanol for 10 minutes [37]. The tissue was rinsed 3x10 min in PBS-TX, and a final 10 min in PBS before being mounted onto slides, allowed to air dry and coverslipped with ProLong^®^ Diamond Antifade Mountant with DAPI (Life Technologies, Carlsbad, CA). Slides were left to cure overnight in the dark, sealed with clear nail polish and imaged over the next 5 days. While not in use, slides were stored in a slide box kept at 4°C.

### Imaging and analysis

Images were captured using the 60x objective on an Olympus FV10i automated confocal laser scanning microscope at the Centre for Microscopy and Cell Imaging, Concordia University, Montreal, Canada. Brain regions of interest were determined based on landmarks from “Brain maps: structure of the rat brain” [38], with the exception of the nucleus accumbens, where landmarks were identified from “The Rat Brain In Stereotaxic Coordinates” [34]. One image for each brain area was taken per slice, up to a maximum of seven slices. For most brain regions, this was sufficient to include all slices containing the area of interest. In larger areas, such as the dorsal striatum and the olfactory tubercle, slices were selected randomly and the dorsal-ventral (or medial-lateral) coordinates were shifted from image to image. Images captured an area of 212 x 212 μm, at depth intervals of 0.8 μm and a resolution of 1024 x 1024 pixels. Laser intensity was set automatically, and then adjusted for each brain area for optimal visualization of fluorescent labeling.

Images were counted using ImageJ freeware (http://imagej.nih.gov). All positive cells regardless of intensity were manually identified in each channel and marked layer-by-layer; each marked cell was superimposed onto every layer so as not to re-count cells that appeared in subsequent layers. A comparison with DAPI was used to define neurons based on the distinct, large, round shape of their nuclei. A composite of the markings in each channel was created, and using the central position of every marking, we were able to extract 1) the total number of neurons as defined by DAPI-stained nuclei, 2) the number of neurons expressing either BMAL1 or PER2, 3) the number of neurons co-expressing Enk or SubP, and 4) the number of neurons expressing Enk or SubP, but not BMAL1 or PER2. The numbers of cells for each image were converted to a percentage of total neurons counted and imported into Prism (Version 6, GraphPad, San Diego, CA) for descriptive statistics and statistical testing. Differences were assessed using two-tailed, unpaired t-tests, except when examining all four markers, where overall distribution was analyzed with a Fisher’s exact test. Levels of expression for each neuropeptide were expected to be different and unique to each brain area examined. Therefore, each brain region was expected to have co-expression levels completely independent of other regions examined. For this reason, each brain area was analyzed separately without the need for adjustments for multiple comparisons. Results presented here are displayed as the mean ± standard error of the mean (SEM). Significance was set at p<0.05.

## Results

BMAL1 and PER2 displayed robust expression in most brain regions imaged. As expected, BMAL1 and PER2 immunoreactivity was mostly nuclear with some cytosolic expression, as evidenced by colocalization with the nuclear DAPI labeling. Enk and SubP were mostly cytosolic and highly enriched in neuronal processes; however only neurons with labeled cell bodies were counted. BMAL1 and PER2 were clearly expressed in both Enk- and SubP-positive cells in brain regions containing these peptides. Moreover, BMAL1 and PER2 were expressed in the majority of identified neurons in nearly all of the brain regions examined.

### The dorsal and ventral striatum

**Fig 1:**
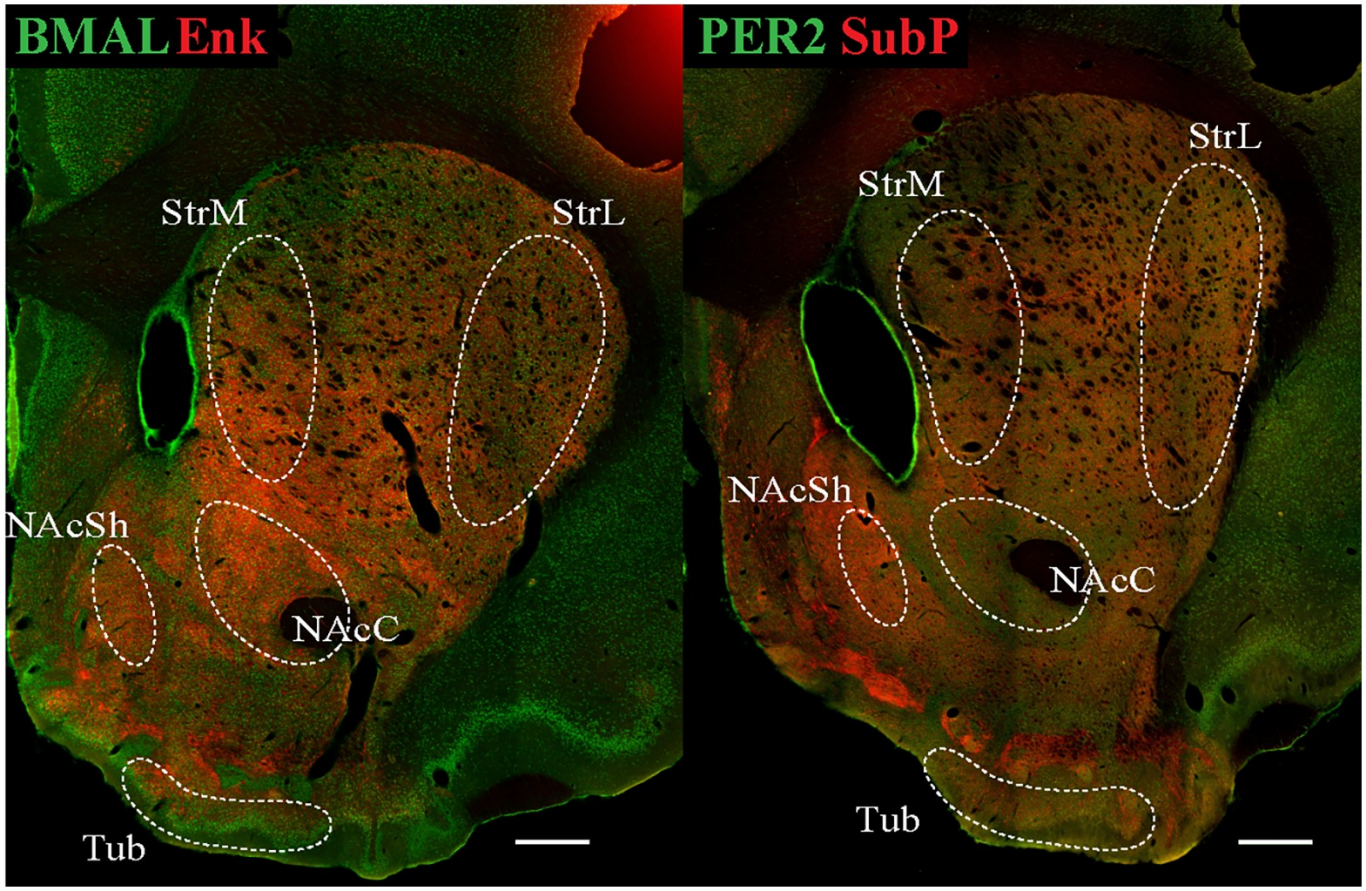
Outline of regions imaged in the dorsal and ventral striatum. Low-magnification confocal laser scanning micrographs of sections stained with antibodies against BMAL1/Enk (left panel) and PER2/SubP (right panel). White dotted lines denote the target sampling regions where images were collected from. Individual confocal images were stitched together to create the single image. Scale bar: 500 μm. StrM, medial dorsal striatum; StrL, lateral dorsal striatum; NAcC, nucleus accumbens core; NAcSh, nucleus accumbens shell; Tub, olfactory tubercle.

The coexpression of PER2 and BMAL1 with Enk or SubP was first analyzed in the dorsal and ventral striatum, including the medial and lateral dorsal striatum (StrM and StrL), nucleus accumbens core and shell (NAcC and NAcSh) and olfactory tubercle (Tub). Example regions where images were taken are shown in Fig 1. In this area of the brain Enk and SubP correspond to D2- and D1-receptor bearing medium spiny neurons respectively [22, 23].

**Fig 2:**
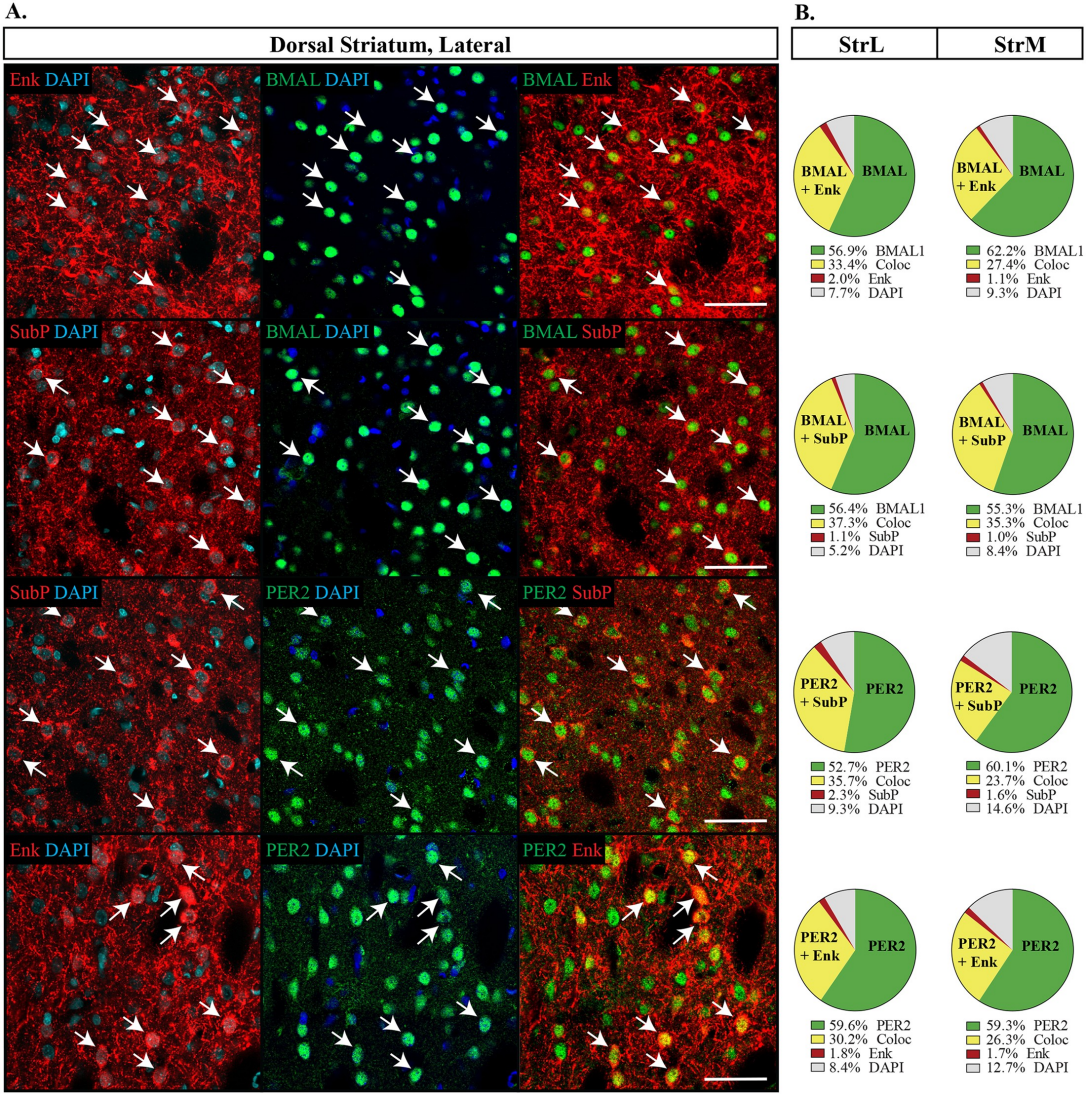
Distribution of proteins in the dorsal striatum. (A) Each combination of antibodies analyzed are shown, sampled from the lateral dorsal striatum (StrL). Enkephalin (Enk) or Substance P (SubP) in red, DAPI in blue and PER2 or BMAL1 (BMAL) in green. Arrows point to the same cell within each row and indicate that clock genes are expressed with both Enk and SupP. Scale bar: 50 μm. (B) Pie charts representing the proportion of cells labelled with each double labeling combination in the StrL or medial dorsal striatum (StrM). The proportion of co-expression of clock genes with BMAL1 or PER2 and SubP or Enk is similar across each possible combination. Green: PER2 or BMAL with DAPI only; Yellow: PER2 or BMAL1 with Enk or SubP (Coloc), representing cells with co-expression; Red: Enk or SubP with DAPI only; Grey: cells identified with DAPI but not labelled with PER2, BMAL, Enk or SubP.

Between the StrM and StrL, expression patterns of all four proteins were similar (Fig 2). Both BMAL1 and PER2 were homogenously expressed throughout the dorsal striatum, though PER2 labelled a slightly lower percentage of cells than BMAL1 (StrM: BMAL1 90.1 **±**0.8%, PER2 85.0 **±**1.2%, p = 0.003; StrL: BMAL1 92.4 **±**0.7%, PER2 89.0 **±**0.7%, p = 0.002). Enk and SubP each labelled about a third of the neurons (Enk: StrM 28.8 **±**1.6%, StrL 34.7 **±**1.6%, SubP: StrM 31.3 **±**1.9%, StrL 34.7 **±**1.5%) and almost always occurred with PER2 or BMAL1 (Fig 2). Furthermore, PER2 and BMAL1 were not preferentially co-expressed with either Enk or SubP (Fisher’s exact test, p = 0.75 StrL; p = 0.48 StrM).

**Fig 3:**
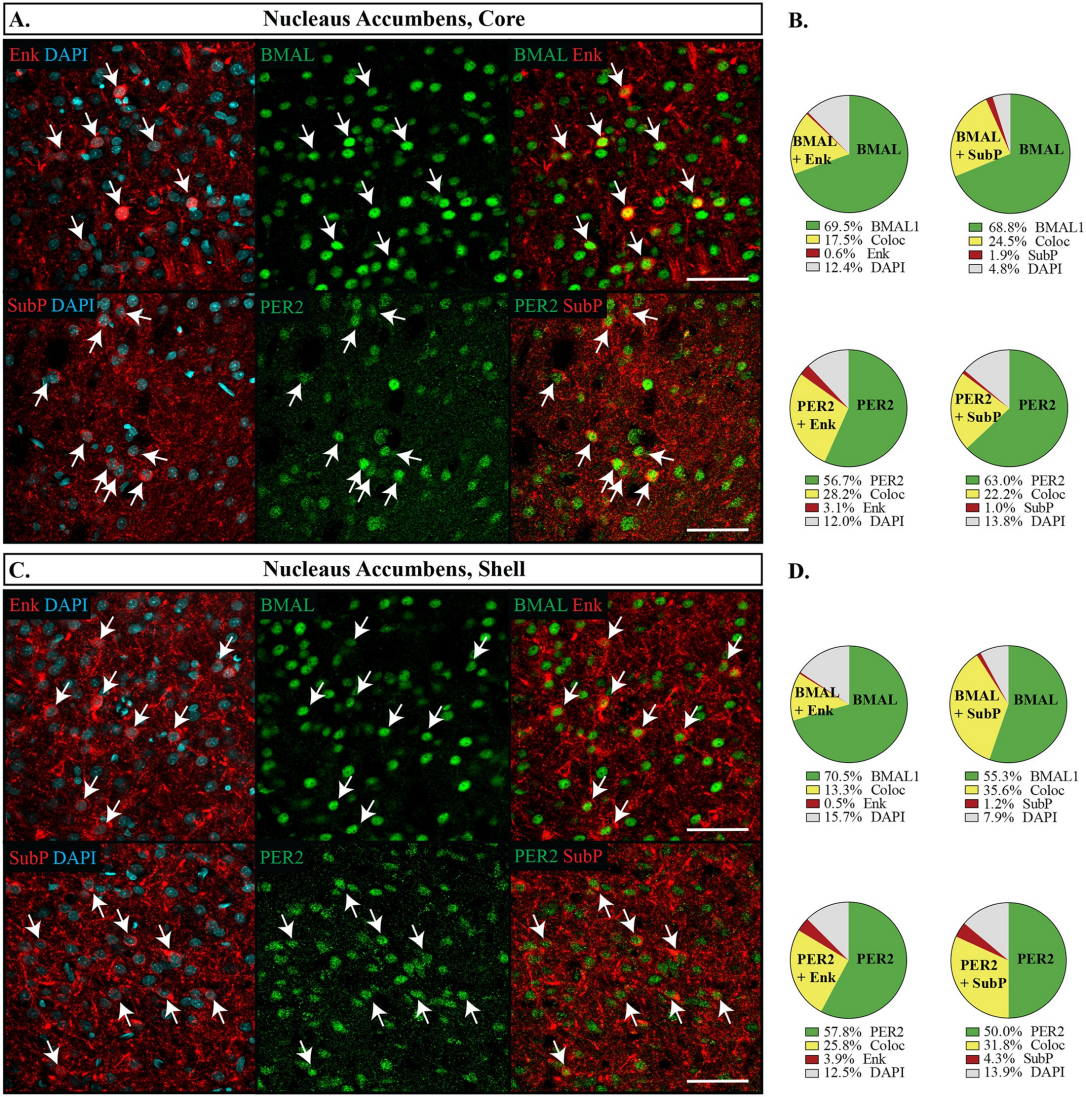
Distribution of proteins in the nucleus accumbens. Immunofluorescent images from the NAcC (A) and NAcSh (C): Enkephalin (Enk) or Substance P (SubP) are shown in red, DAPI in blue and PER2 or BMAL1 (BMAL) in green. Arrows point to cells labelled with both proteins and indicate the same cell across the row. Scale bar: 50 μm. Pie charts representing the proportion of each labelled cell combination counted in the core (B) and shell (D). Green: PER2 or BMAL1 with DAPI only, Yellow: proportion co-expressing PER2 or BMAL1 with Enk or SubP (Coloc), Red: Enk or SubP with DAPI only, Grey: cells identified with DAPI but not labelled with PER2, BMAL1, Enk or SubP.

**Fig 4:**
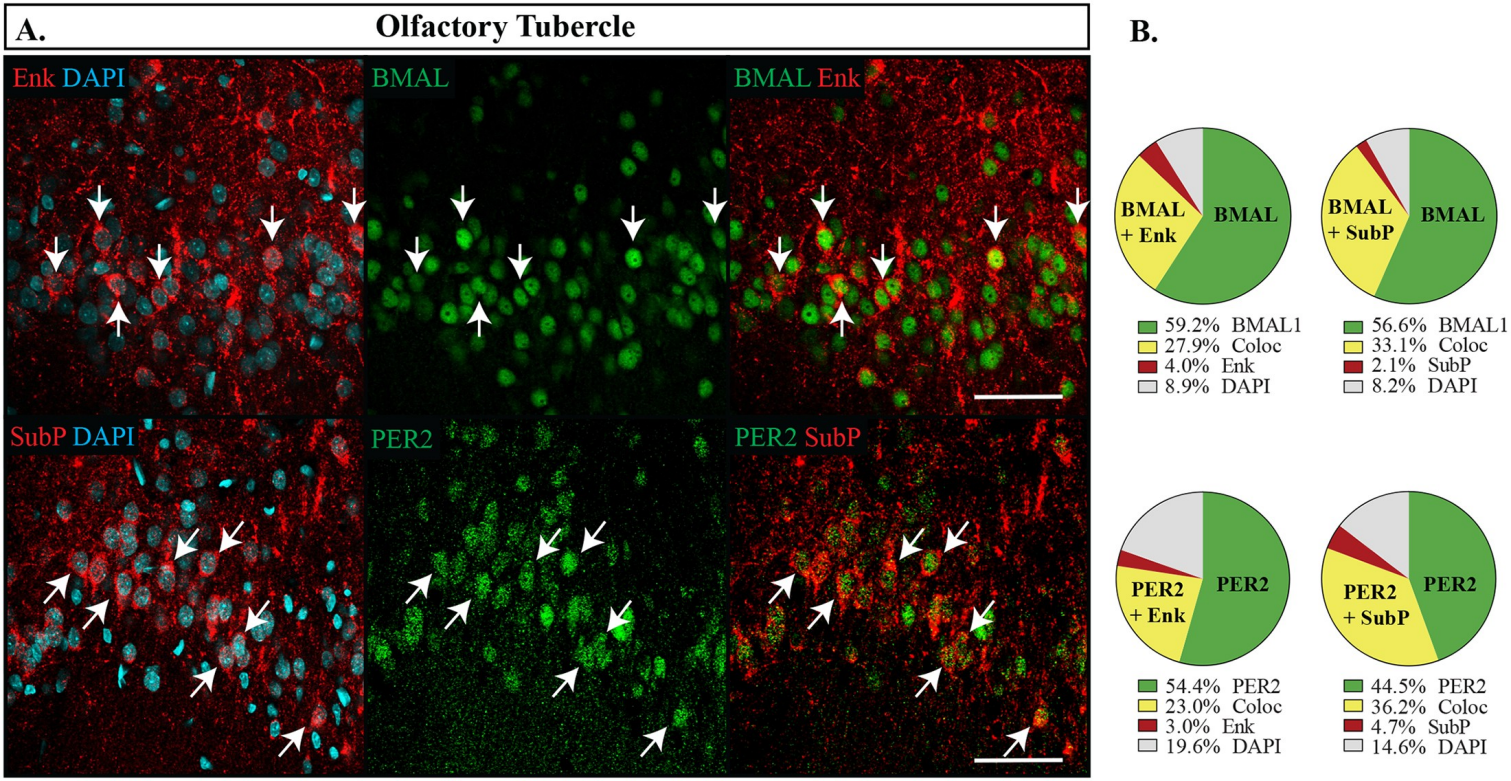
Distribution of proteins in the olfactory tubercle. (A) Localization of proteins in the olfactory tubercle. Enkephalin (Enk) or Substance P (SubP) are shown in red, DAPI in blue and PER2 or BMAL1 (BMAL) in green. Arrows point to cells labelled with both proteins and indicate the same cell across the row. Scale bar: 50 μm. (B) Pie charts representing the proportion of each labelled cell combination. Green: PER2 or BMAL1 with DAPI only, Yellow: co-expression of PER2 or BMAL1 with Enk or SubP (Coloc), Red: Enk or SubP with DAPI only, Grey: cells identified with DAPI but not labelled with PER2, BMAL1, Enk or SubP.

In the nucleus accumbens, BMAL1 and PER2 were uniformly expressed in most neurons at similar levels as the dorsal striatum (NAcC: BMAL1 88.8 **±**1.6%, PER2 84.9 **±**1.8%; NAcSh: BMAL1 86.7 **±**1.7%, PER2 82.9 **±**1.6%). However, the expression patterns of Enk and SubP differed slightly. As reported elsewhere for D1- and D2-receptor expression [39], SubP and Enk appeared rather uniformly in the NAcC, but had a patchier appearance in the NAcSh. Images were sampled from the medial region of the shell because it had relatively high expression of both Enk and SubP (see Fig 1). Nearly all Enk and SubP expressing cells also expressed BMAL1 or PER2 in both the core and the shell (Fig 3), with no preference given for either cell type (Fisher’s exact test, p = 0.06 NAcC, p = 0.10 NAcSh), however, a lower percentage of cell bodies overall were counted expressing Enk in the shell (Enk 22.7 **±**1.9%, SubP 36.4 **±**1.8%, p<0.001). The pattern of expression from the NAcSh was extended to the Tub (Fig 4), which also had overall lower expression levels of Enk (Enk 28.6 **±**1.6%, SubP 41.1 **±**2.7%, p = 0.001) and lower expression of PER2 (PER2 82.4 **±**1.7%, BMAL1 90.1 **±**1.2%, p = 0.001), however, PER2 and BMAL1 were still not preferentially expressed with either Enk or SubP (Fisher’s exact test, p = 0.73).

### Mesocortical dopamine projection regions

The mesocortical dopamine system projects throughout the limbic forebrain to regions that contribute largely to behaviours including motivation, emotion and long term memory. Among others, they include the central nucleus of the amygdala (CEA), the basolateral amygdala (BLA), the oval nucleus and the principal nucleus of the bed nucleus of the stria terminalis (BNSTov and BNSTp) and the hippocampus (Hip) [40]. The CEA and BNSTov share similar anatomical and functional properties and are often referred to together as the central extended amygdala [41]. These two nuclei appear to be unique in their clock gene rhythm regulation. They peak in antiphase to the other limbic forebrain regions [6], and interestingly, the BNSTov, but not the CEA, is affected by global dopamine depletion [21], suggesting that there may be different entrainment mechanisms for these two nuclei. The two main neuronal populations of the CEA and BNSTov produce either Enk or corticotropin-releasing hormone (CRH) [24].

**Fig 5:**
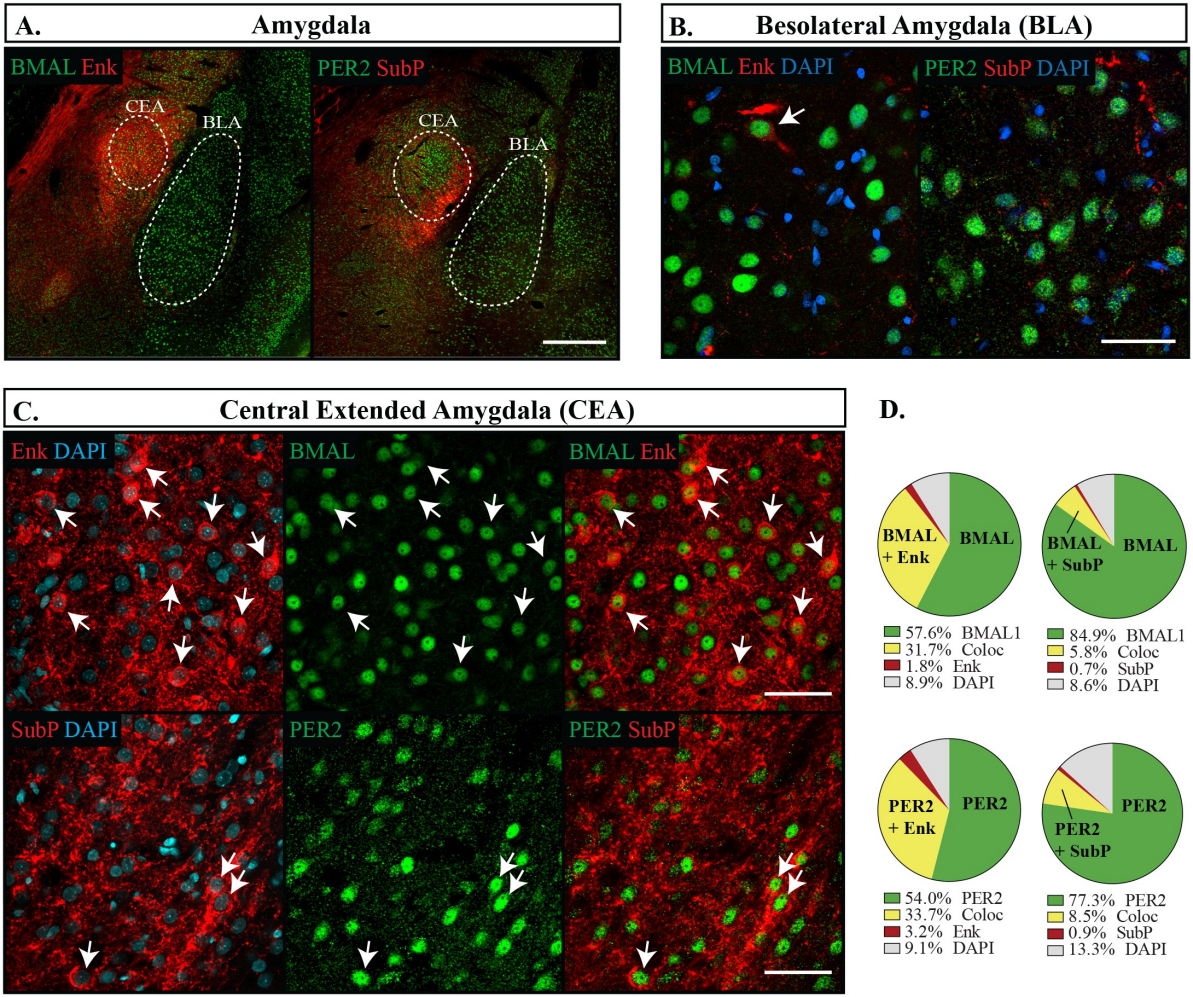
Distribution of proteins in the amygdala. (A) Low magnification images of the sampling regions from the central nucleus of the amygdala (CEA) and basolateral amygdala (BLA) are denoted by the white dotted line. Scale bar: 500 μm. (B&C) High magnification images showing localization of proteins in the BLA (B), or the CEA (C) showing PER2 or BMAL1 (green), Enk or SubP (red), DAPI (blue). Arrows point to cells labelled with both proteins and indicate the same cell across a row. Scale bar: 50 μm. (D) Pie charts representing the proportion of cells with each possible labelling combination counted in the CEA. Green: PER2 or BMAL1 with DAPI only, Yellow: PER2 or BMAL1 co-expressing Enk or SubP (Coloc), Red: Enk or SubP with DAPI only, Grey: cells identified with DAPI but not labelled with PER2, BMAL1, Enk or SubP.

**Fig 6:**
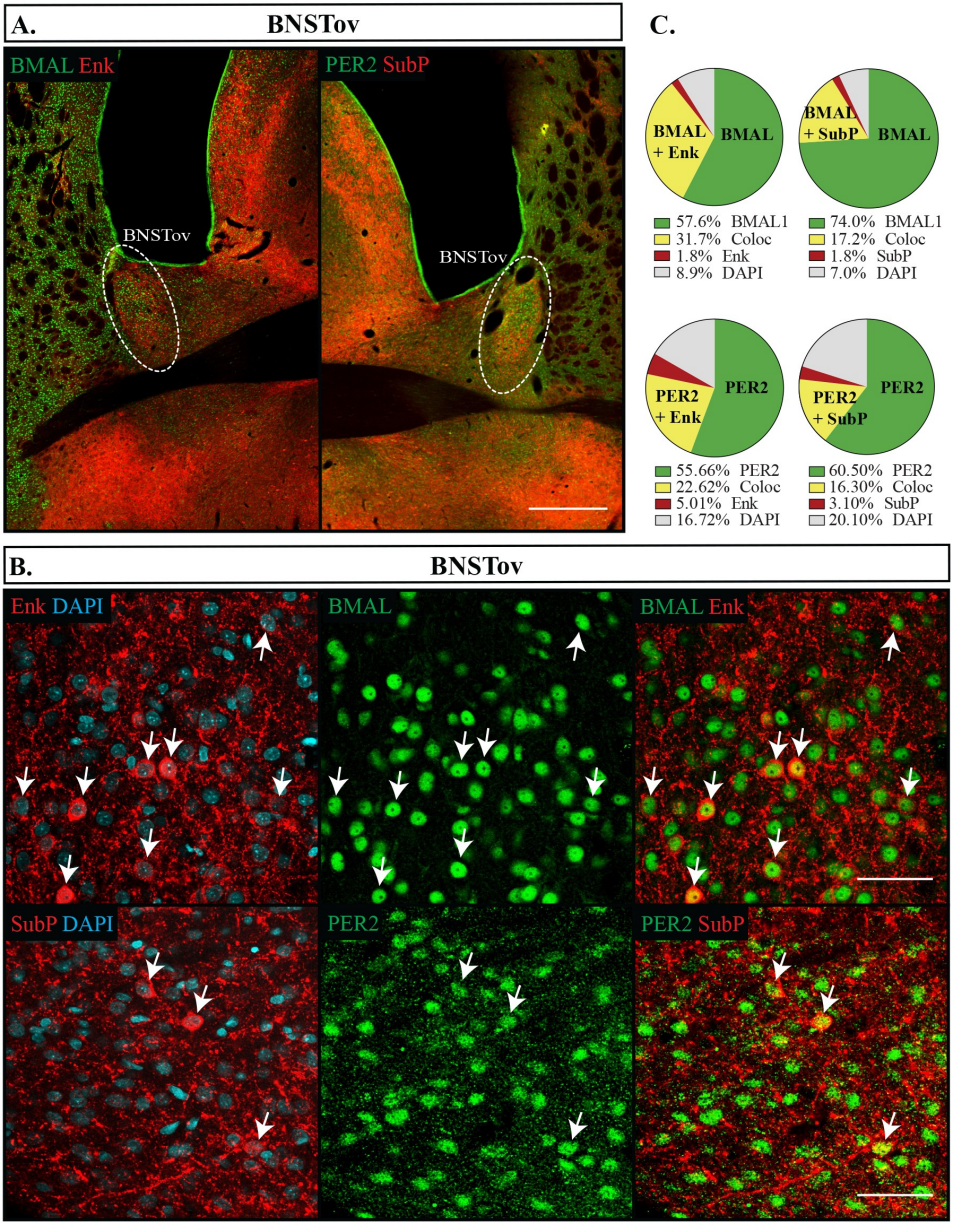
Distribution of proteins in the BNSTov. (A) Outlines of regions imaged in the oval nucleus of the bed nucleus of the stria terminalis (BNSTov) are shown. Scale bar: 500 μm. (B) Localization of proteins in the BNSTov showing PER2 or BMAL1 (green), Enk or SubP (red), DAPI (blue). Arrows point to cells labelled with both proteins and indicate the same cell across the row. Scale bar: 50 μm. (C) Pie charts representing the proportion of each labelled cell combination in the BNSTov. Green: PER2 or BMAL1 with DAPI only, Yellow: PER2 or BMAL1 co-expressing Enk or SubP (Coloc), Red: Enk or SubP with DAPI only, Grey: cells identified with DAPI but not labelled with PER2, BMAL1, Enk or SubP.

**Fig 7:**
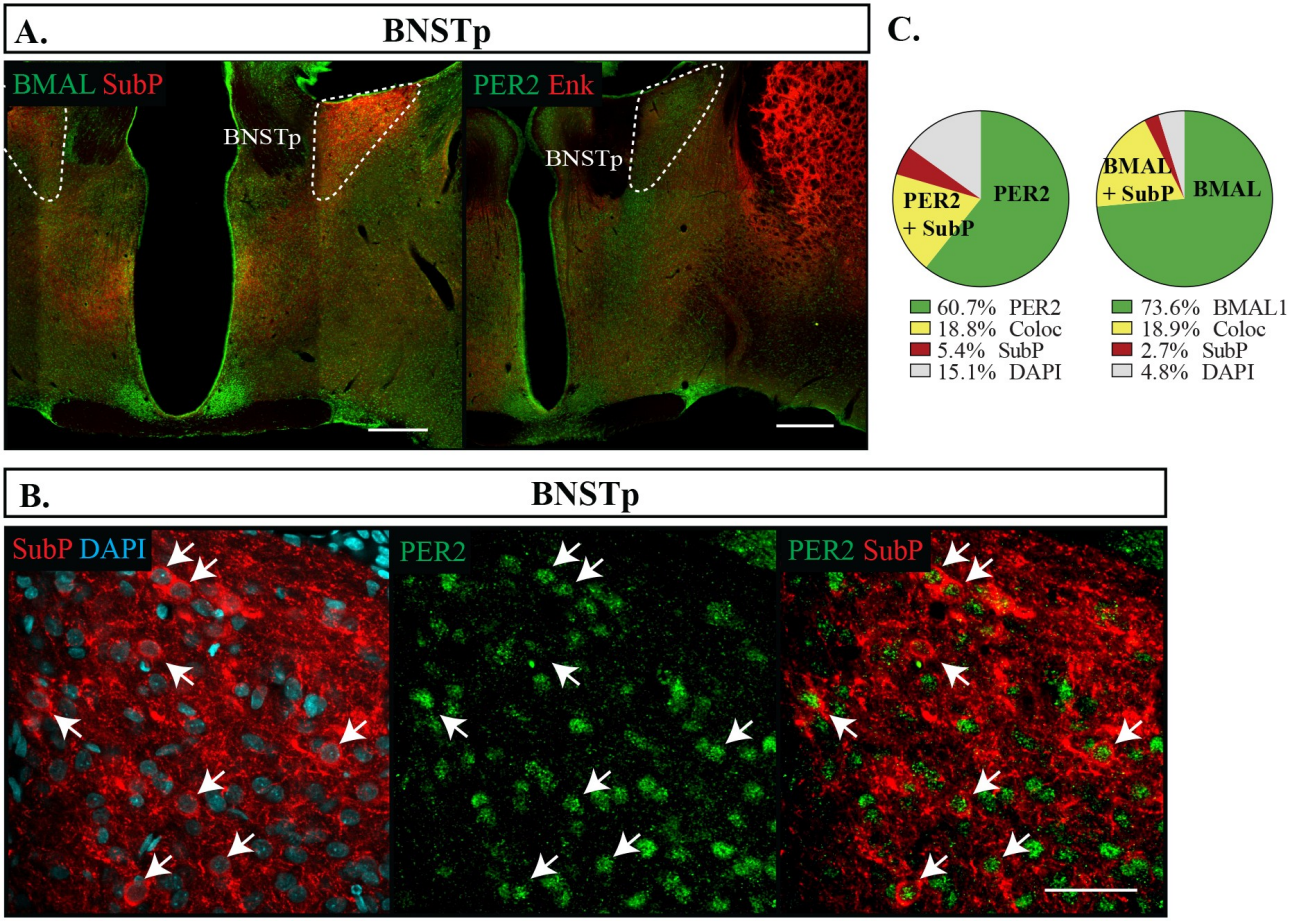
Distribution of proteins in the BNSTp. (A) Outlines of regions imaged in the principal nucleus of the bed nucleus of the stria terminalis (BNSTp) are shown. Scale bar: 500 μm. (B) Localization of proteins in the BNSTp showing PER2 or BMAL1 in green, Enk or SubP in red, and DAPI in blue. Arrows point to cells labelled with both proteins and indicate the same cell across a row. Scale bar: 50 μm. (C) Pie charts representing the proportion of each labelled cell combination in the BNSTp. Green: PER2 or BMAL1 with DAPI only, Yellow: PER2 or BMAL1 co-expressing SubP (Coloc), Red: SubP with DAPI only, Grey: cells identified with DAPI but not labelled with PER2, BMAL1, Enk or SubP.

**Fig 8:**
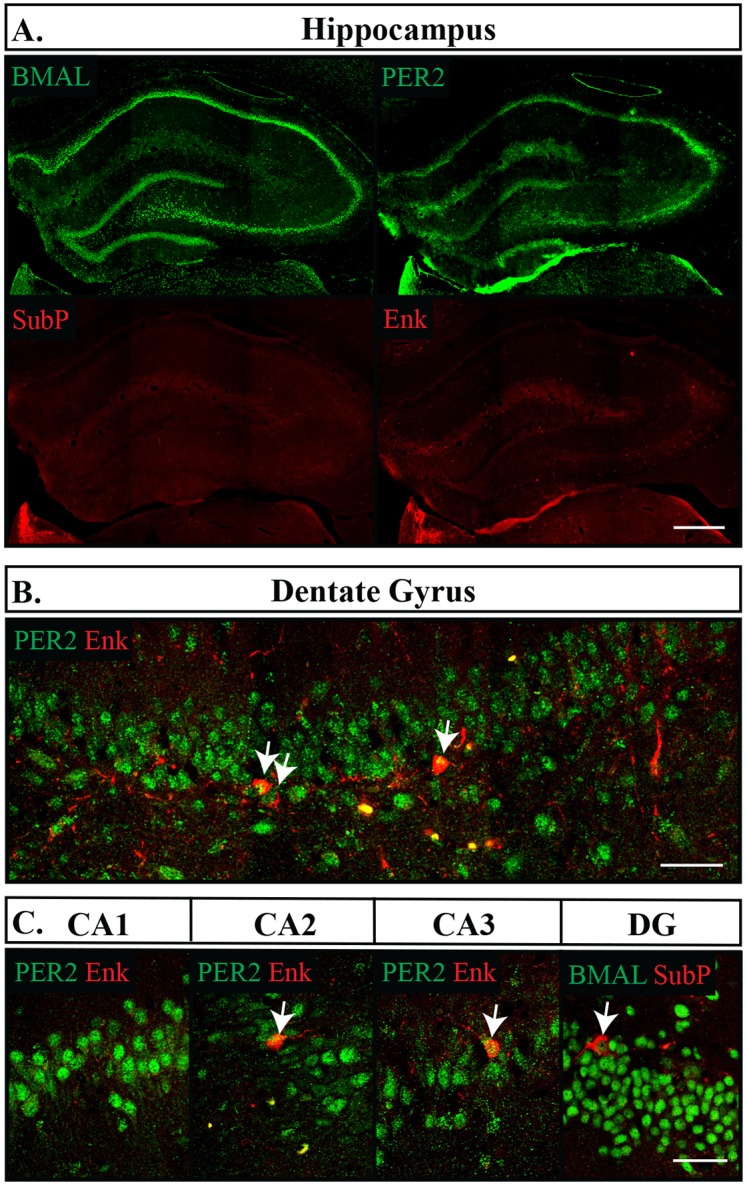
Distribution of proteins in the hippocampus. (A) Subsections of the hippocampus are shown. Top left, BMAL1; top right, PER2; bottom left, Substance P (SubP); bottom right Enkephalin (Enk). Scale bar: 500 μm. (B) PER2 (green) and Enk (red) imaged in the dentate gyrus (DG) of the hippocampus. (C) PER2 or BMAL1 (green) and Enk or SubP (red) in regions CA1, CA2, CA3 and the dentate gyrus. Arrows point to cells labelled with both proteins. Scale bar: 50 μm.

We found that the CEA and BNSTov had similar expression patterns of Enk, but differed slightly in their expression levels of SubP. Enk labelled about a third of cells (CEA: 35.3 **±**1.8%, BNSTov: 26.7 **±**1.3%), while in both regions a considerably smaller proportion of cells were labelled with SubP, with only 8.2% **±**0.7% showing immunoreactivity in the CEA and 18.8% **±**1.8% in the BNSTov. Nonetheless, Enk and SubP were nearly always co-expressed with both BMAL1 and PER2, and BMAL1 and PER2 continued to be expressed in nearly all neurons, however PER2 was expressed at lower levels that BMAL1 (CEA: BMAL1 90.2 **±**0.9%, PER2 86.7 **±**0.9%, p = 0.013; BNSTov: BMAL1 91.7 **±**0.8%, PER2 78.1 **±**1.9%, p = 0.001) (Figs 5 and 6). Neither BMAL1 nor PER2 showed an association with either peptide (Fisher’s exact test, p = 0.68). The BNSTp showed a slightly different expression profile than the BNSTov, and had nearly a third of its cells labelled with SubP (22.3 **±**1.9%) but no cell bodies were found labelled with Enk. Nonetheless, BMAL1 was still present in the majority of neurons and PER2 trended to a slightly lower proportion (BMAL1 90.5 **±**2.2%, PER2 78.0 **±**3.4%, p = 0.059) (Fig 7). In addition, SubP-positive cells were consistently found with both clock proteins (Fig 7). In the Hip (Fig 8) and the BLA (Fig 5), Enk and SubP were only occasionally labelled (<1% of cells) and were thus not analyzed further.

### Thalamic and hypothalamic nuclei

**Fig 9:**
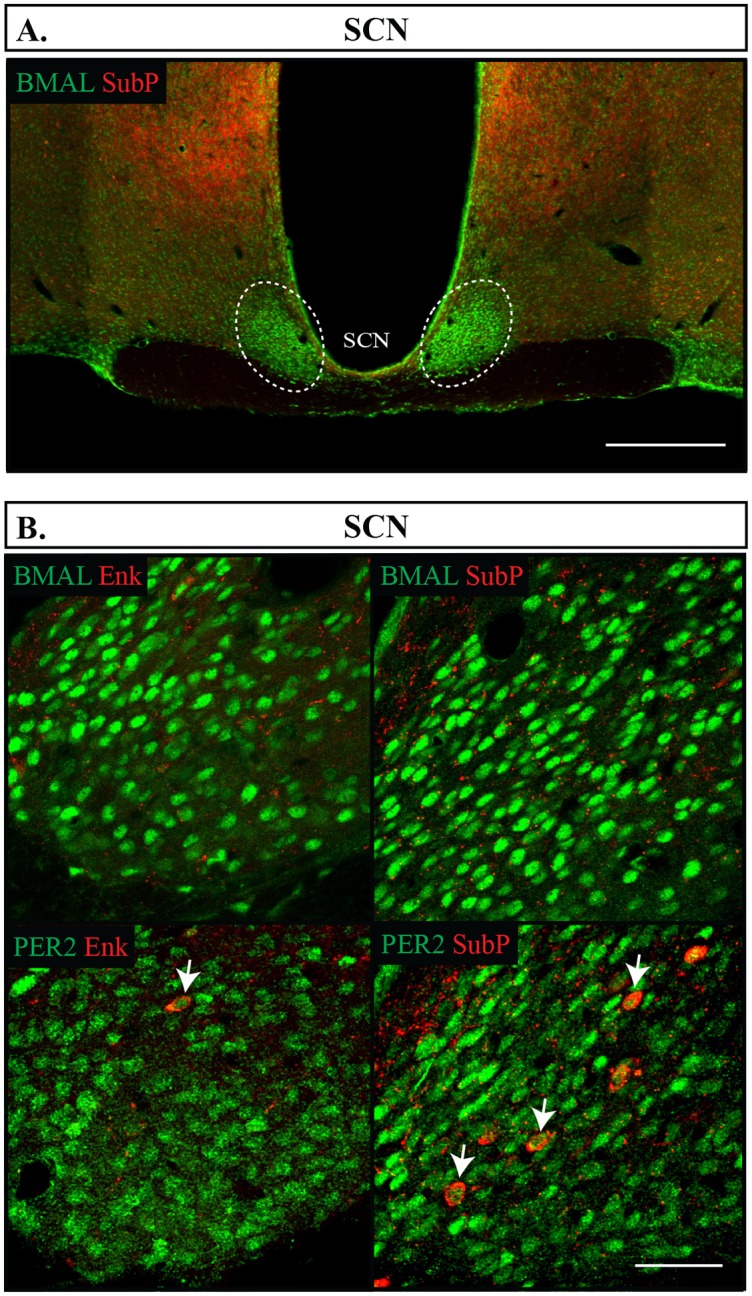
Distribution of proteins in the SCN. (A) Outlines of the region imaged in the SCN are shown. Scale bar: 500 μm. (B) Each combination of protein labelled in the SCN is illustrated, BMAL1 and PER2 in green, Enk or SubP in red. Arrows point to single cells labelled with both proteins. Scale bar: 50 μm.

The SCN was only occasionally labelled with SubP or Enk (<1%) (Fig 9), as has been reported elsewhere [42], and was therefore not analyzed further. A major target of the SCN is the hypothalamic paraventricular nucleus (PVH). Neurosecretory cells in the medial and ventral PVH secrete regulatory hormones such as CRH and TRH to the median eminence where they act upon the anterior pituitary and regulate the neuroendocrine system [43]. Enk is expressed in a subset of neurons that partially overlaps with expression of CRH, TRH and oxytocin [25–27]. Another direct target of the SCN is the arcuate nucleus in the hypothalamus (Arc), which regulates nutrient intake and energy balance. There are two populations of neurons in the Arc: 1) Neuropeptide Y and Agouti-related peptidic (NPY/AgRP) neurons associated with increased food intake and anabolism and 2) anorexic and catabolic related neurons that express pro-opiomelanocortin (POMC) [44]. Both Enk and SubP are likely expressed in the NPY/AgRP set of neurons [45].

**Fig 10:**
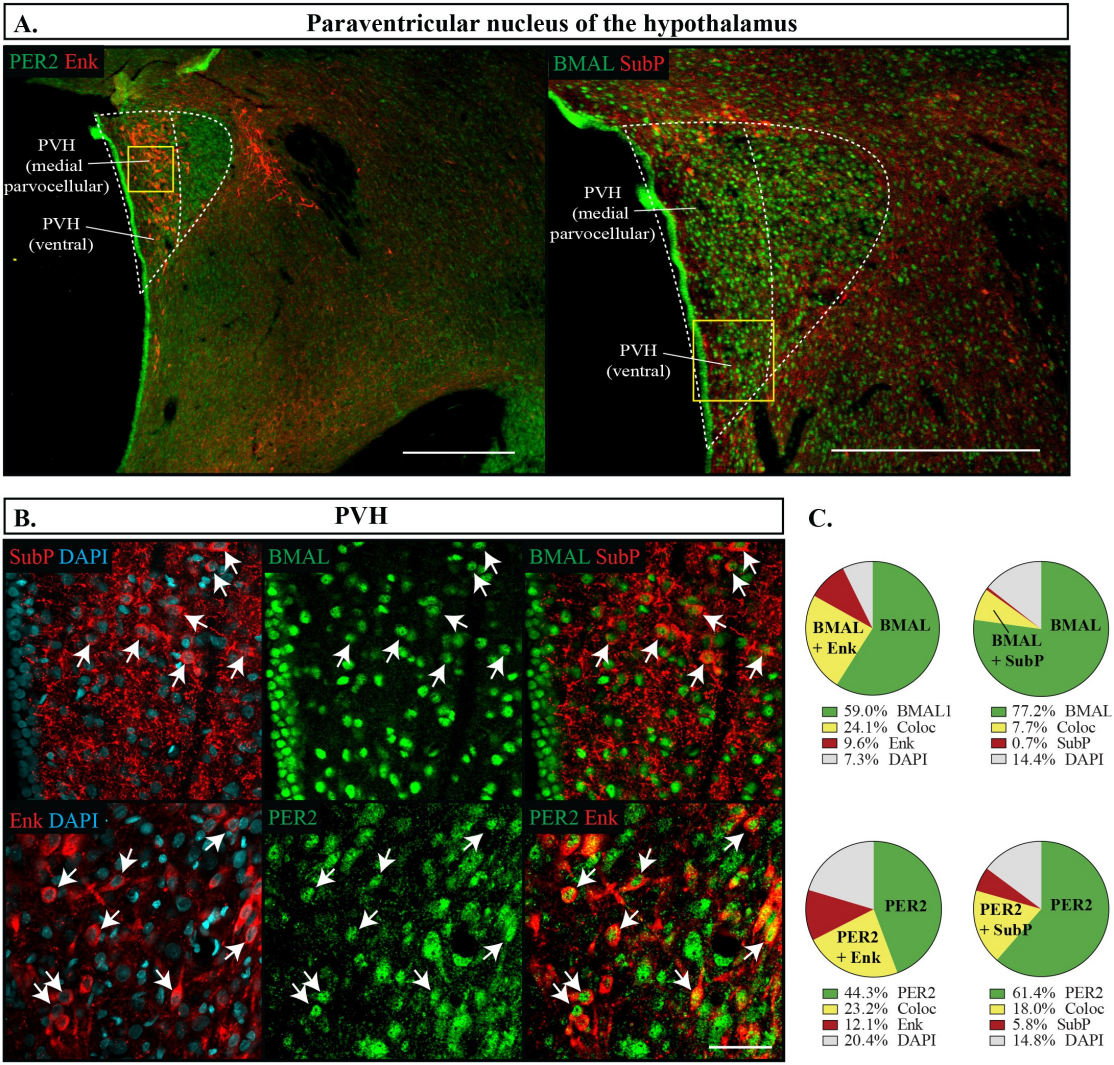
Distribution of proteins in the paraventricular nucleus. (A) Outlines of regions imaged in the paraventricular nucleus of the hypothalamus (PVH) are shown to the left of the dashed white line. Scale bar: 500 μm. The yellow box represents the region from where the below images were sampled from. (B) Localization of proteins in the PVH showing PER2 or BMAL1 in green, Enk or SubP in red, and DAPI in blue. Arrows point to cells labelled with both proteins and indicate the same cell across the row. Scale bar: 50 μm. (C) Pie charts representing the proportion of each labelled cell combination in the PVH. Green: PER2 or BMAL1 with DAPI only, Yellow: PER2 or BMAL1 co-expressing Enk or SubP (Coloc), Red: Enk or SubP with DAPI only, Grey: the remaining cells identified with DAPI but not labelled with PER2, BMAL1, Enk or SubP.

**Fig 11:**
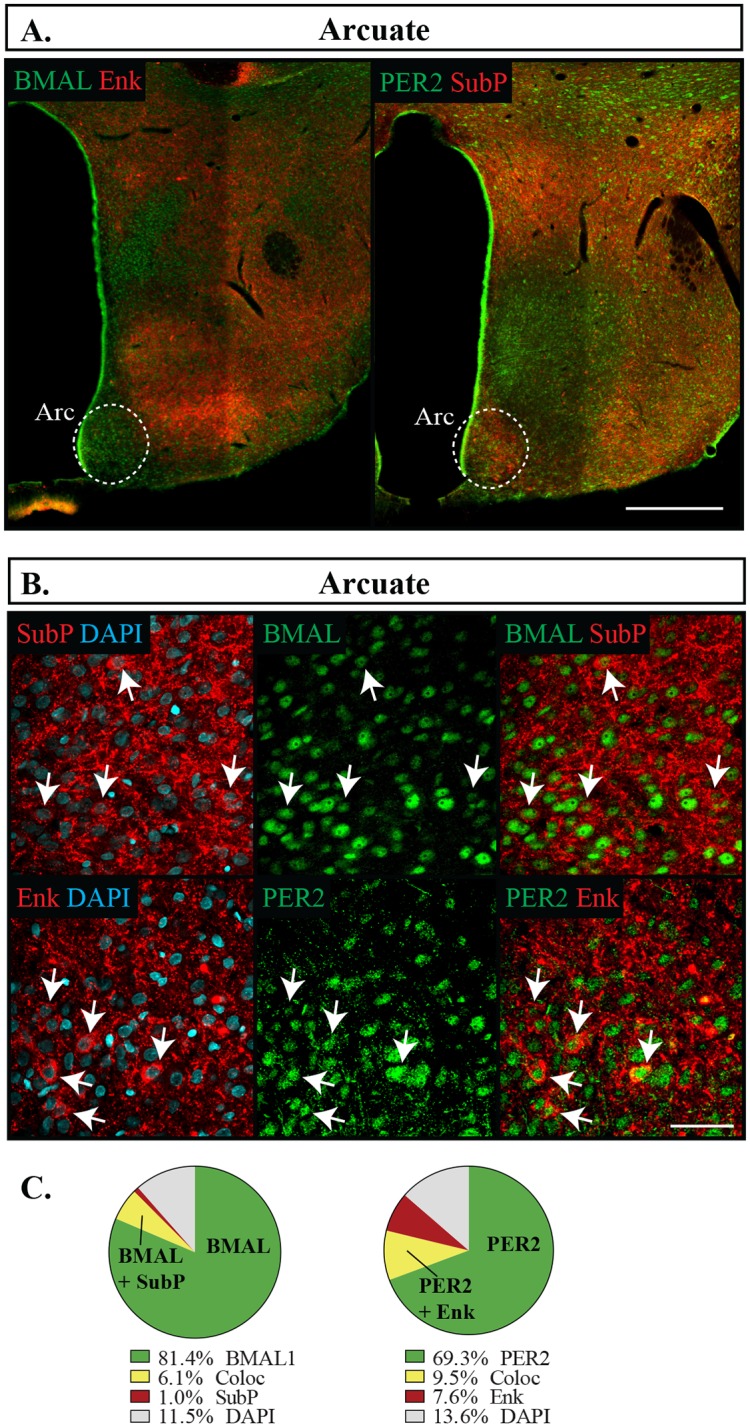
Distribution of proteins in the arcuate nucleus. (A) Outlines of regions imaged in the arcuate nucleus (Arc). Scale bar: 500 μm. (B) Localization of proteins in the Arc showing PER2 or BMAL1 in green, Enk or SubP in red, and DAPI in blue. Arrows point to cells labelled with both proteins and indicate the same cell across the row. Scale bar: 50 μm. (C) Pie charts representing the proportion of each labelled cell combination in the Arc. Green: PER2 or BMAL1 with DAPI only, Yellow: PER2 or BMAL1 co-expressing Enk or SubP (Coloc), Red: Enk or SubP with DAPI only, Grey: cells identified with DAPI but not labelled with PER2, BMAL1, Enk or SubP.

In the PVH, BMAL1 and PER2 were well labelled in the medial and ventral parvocellular region, however at slightly lower proportions than in the limbic forebrain, with PER2 being expressed in a lower proportion of cells than BMAL1 (BMAL1 83.6 **±**1.6%, PER2 72.6 **±**2.3%, p = 0.001). In the PVH, SubP was localized more in the ventral region and was almost always co-expressed with PER2 or BMAL1, whereas Enk was located throughout these regions and had a much higher proportion of cells labelled with Enk independent of BMAL1 and PER2 (Fig 10). Unlike the previous regions analyzed, about one-third of the cells labelled with ENK were not co-expressed with BMAL1 or PER2. As has been reported elsewhere, SubP and Enk were relatively sparse in the Arc with 14.5 **±** 1.4% cells expressing Enk and 15.1 **±**4.2% expressing SubP [45, 46]. However, this nucleus also had a higher proportion of cells that were labelled with Enk but lacked PER2 or BMAL1 immunoreactivity (Fig 11). Still, neither PER2 or BMAL1 were favourably expressed with either SubP or Enk (Fisher’s exact test, p = 0.06). In this region, PER2 was also expressed in a lower percentage of cells than BMAL1 (BMAL1 85.5 **±**1.2%, PER2 78.7 **±**1.2%, p = 0.004).

**Fig 12:**
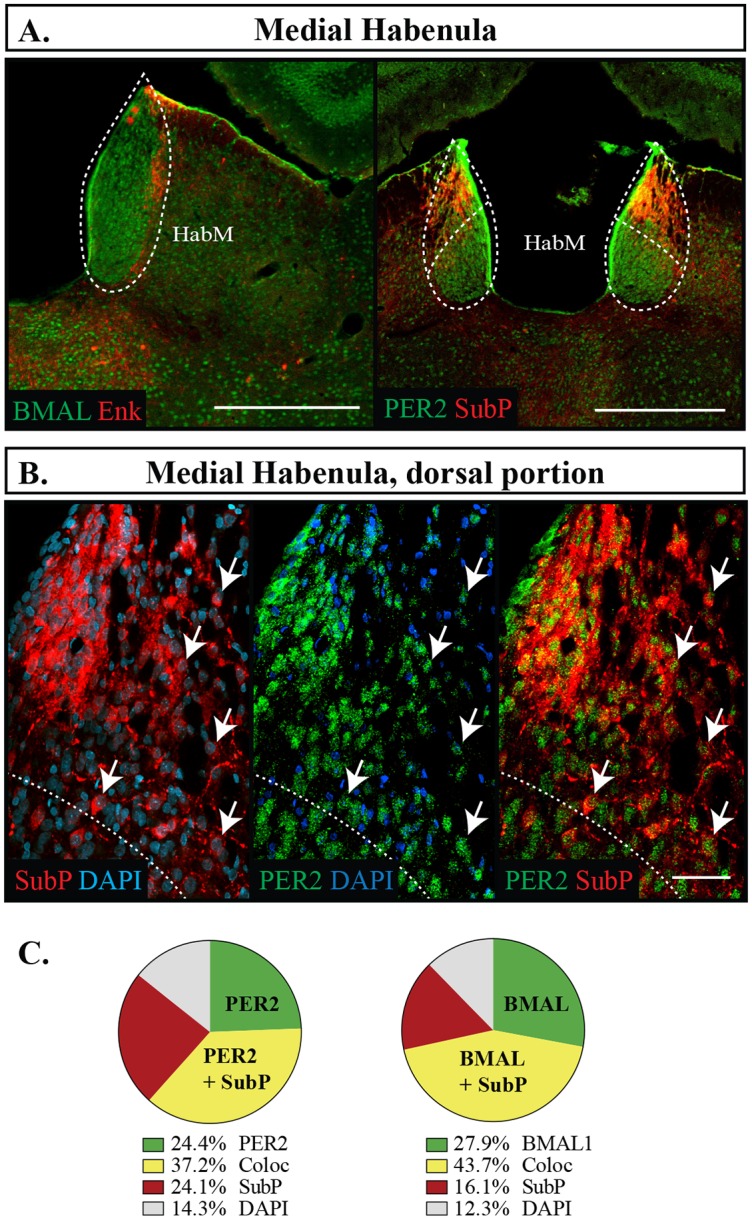
Distribution of proteins in the medial habenula. (A) Outlines of regions imaged in the medial habenula (HabM). Scale bar: 500 μm. (B) Localization of proteins in the dorsal portion on the HabM showing PER2 or BMAL1 in green, SubP in red, and DAPI in blue. Arrows point to cells labelled with both proteins and indicate the same cell across the row. Scale bar: 50 μm. Only the region above the dashed line were counted. (C) Pie charts representing the proportion of each labelled cell combination in the dorsal portion of the HabM. Green: PER2 or BMAL1 with DAPI only, Yellow: PER2 or BMAL1 co-expressing (Coloc), Red: SubP with DAPI only, Grey: cells identified with DAPI but not labelled with PER2, BMAL1, or SubP.

The habenula is located in the dorsal thalamus and can be divided into two segments, the lateral habenula (HabL), which is often associated with limbic function, and the medial habenula (HabM), which is associated with motor and neuroendocrine function [32, 47]. No cell bodies were identified with Enk or SubP in the HabL, and was therefore not examined. The dorsal region of the HabM is characterized by a high density of cholinergic cells that also express SubP [32]. Of all the regions we analyzed, this region had the highest levels of BMAL1 and PER coexpression with SubP, owing to 52.7 **±**5.8% of its cells being identified with SubP, a proportion that is considerably higher than any other region we assessed (Fig 12). This region also had only moderate expression levels of PER2 and BMAL1 (PER2 71.4 **±**1.4%, BMAL1 61.6 **±**6.5%, p = 0.15), with SubP being located in a partially separate neuronal population, given by the increased proportion of SubP-positive cells lacking either BMAL1 or PER2 (Fig 12C).

### The olfactory bulb

**Fig 13:**
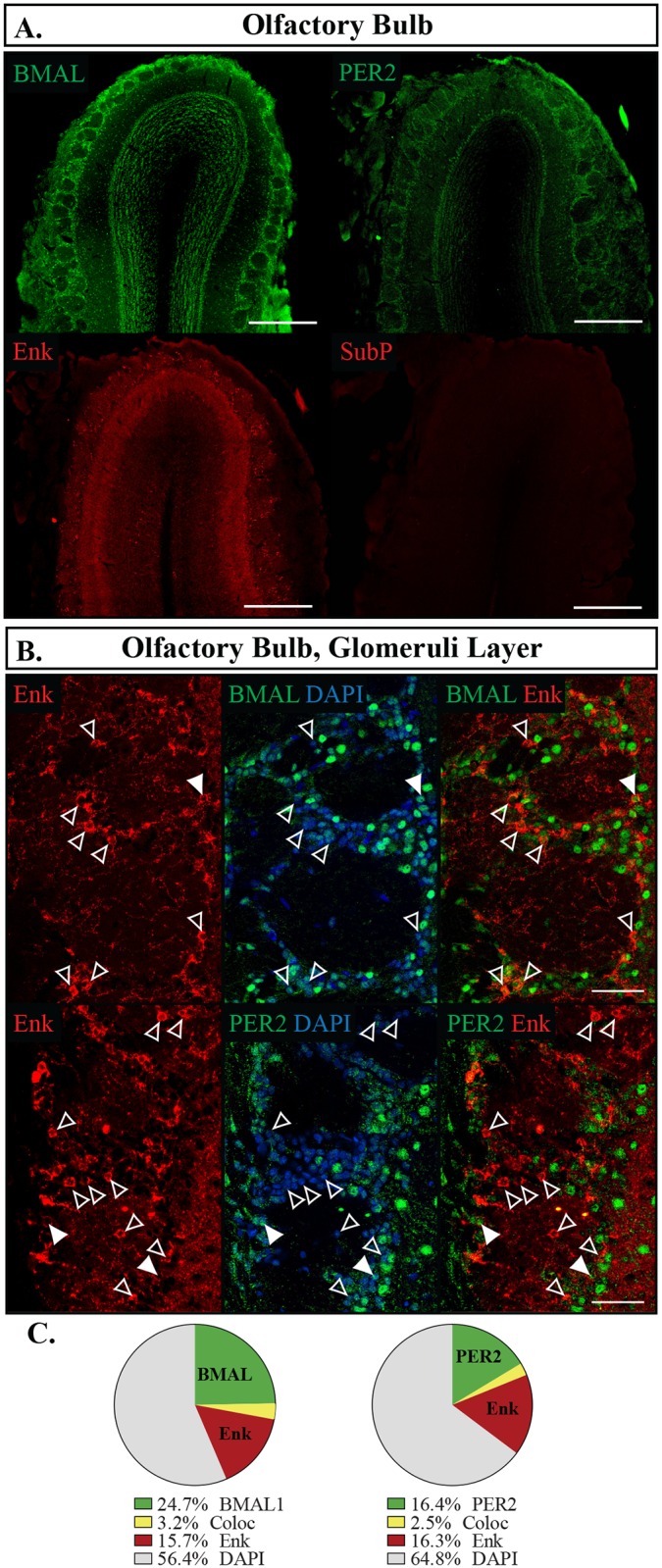
Distribution of proteins in the olfactory bulb. (A) Localization of proteins in the olfactory bulb (OB). Individual confocal images were stitched together to create the single image. Scale bar: 500 μm. Top left, BMAL1; top right, PER2; bottom left, Enkephalin (Enk); bottom right, Substance P (SubP). (B) Localization of proteins in the glomerular layer of the OB showing PER2 or BMAL1 in green, Enk in red, and DAPI in blue. Filled arrows point to cells labelled with both proteins, open arrows point to cells labelled with Enk only. Each arrow indicates the same cell across the row. Scale bar: 50 μm. (C) Pie charts representing the proportion of each labelled cell combination in the glomerular layer of the OB. Green: PER2 or BMAL1 with DAPI only, Yellow: PER2 or BMAL co-expressing Enk (Coloc), Red: Enk with DAPI only, Grey: cells identified with DAPI but not labelled with PER2, BMAL1 or Enk.

Along with the retina, the olfactory bulb (OB) is the only neural structure that can autonomously sustain clock gene rhythms outside of the SCN [48]. It also possesses its own local circuity involving locally produced dopamine [49–51]. In the OB, PER2 was expressed at lower levels than BMAL1, however of all the areas we examined, the OB was unique in that it was the only brain region where BMAL1 and PER2 were only found in a minority of cells (PER2 18.8 **±**1.3%, BMAL1 28.0 **±**3.3%, p = 0.004). Furthermore, BMAL1- and PER2-expressing subpopulations exhibited differing expression profiles. BMAL1 was highly expressed in the granule cell layer and moderately expressed in the glomerular area, whereas PER2 was expressed in the mitral cells and some cells along the inner region of the glomerular layer (Fig 13a). Enk was expressed to some extent throughout the OB, whereas SubP expression was almost nonexistent [Fig 13a]. Enk densely labelled fibres in the granular layer, however cell bodies could only be identified in the glomerular layer, so this was the only region examined for co-expression. Unlike in the other regions analyzed, Enk was primarily expressed independently of PER2 and BMAL1 (Fig 13b and 13c).

## Discussion

In this study we have characterized the coexpression of core circadian clock proteins, BMAL1 and PER2, with the neuropeptides SubP and Enk throughout the rodent forebrain. Apart from the OB, PER2 and BMAL1 were homogenously expressed in the majority of neurons (up to 90%), despite very different expression profiles of SubP or Enk in each nucleus. In nuclei that expressed both SubP and Enk, PER2 and BMAL1 were not preferentially co-expressed in one cell type or the other.

One caveat of this study is that SubP and Enk localize primarily to fibres, and intraventricular colchicine delivery was thus required to improve their visualization within cell bodies. Despite this treatment, fibres remained extensively labelled with Enk and SubP in areas such as the dorsal striatum and nucleus accumbens, sometimes making the identification of cell bodies difficult. For this reason, we estimate that the actual proportion of cells expressing either SubP or Enk to be higher than reported here. For example, in the dorsal striatum, it has been reported that close to 50% of cells express SubP or Enk [52, 53]. Here, we report numbers between 29% and 35%. Nonetheless, the distributions we saw were similar to those reported elsewhere [39, 53]. In addition, since SubP and Enk are nearly equally distributed throughout the dorsal striatum, and since PER2 and BMAL1 were expressed in most cells, we can confidently conclude that these clock proteins are expressed in both cell types.

Colchicine disrupts microtubule polymerization, preventing axonal transport of peptides and is therefore commonly used to visualize secreted peptides within the cells that manufacture them [33, 54, 55]. To the best of our knowledge, this does not affect endogenous expression of circadian proteins. Given that secreted peptides play a role in synchronizing circadian gene expression in the SCN [56], colchicine can render the SCN and many downstream signals arrhythmic [57]. However, this has only been demonstrated after several days, and given the short time frame of 24–36 hours used in the present study, it is unlikely that this would have affected peak expression levels of PER2 or BMAL1.

Here, we have only examined the anatomical localization of BMAL1 and PER2 at the time of peak expression; at the times when we expected the highest number of neurons to have visible clock protein expression. We did not address rhythmicity within these different cell types, which leaves open the question of whether their molecular clocks possess similar rhythmic properties, such as phase and amplitude of clock protein oscillations. Much of what we know about the dynamics of circadian gene expression has been studied in the SCN, but has yet to be explored systematically in downstream brain regions. The SCN contains a heterogeneous mixture of neurons that express a variety of different neuropeptides that function together to form a coherent network [42]. With the advances of genetic and imaging techniques, it is now possible to follow clock gene expression longitudinally in time at a single cell resolution using fluorescent reporter proteins. At the tissue level, PER2 in the SCN appears to reach a peak around 12–14 hours from the beginning of the light phase (ZT12-ZT14) [58, 59] However, between individual neurons, a specific spatiotemporal pattern of expression exists, whereby the peak time of clock gene expression differs slightly from neuron to neuron, creating a wave-like pattern throughout the nucleus over the course of a day [60]. Single neuron imaging techniques have also shown *in-vitro* that amplitudes of PER2 oscillations vary throughout the SCN. Some neurons even remain arrhythmic, expressing near-constant PER2 levels at all times of day [42]. In studying rhythms in single neurons outside of the SCN, Guilding et al. [61] similarly discovered heterogeneity between molecular clocks in downstream regions. Specifically, they found that in the dorsal Arc 89% of neurons identified with a PER2:Luciferase assay were rhythmic, and even fewer, 67%, were rhythmic in the lateral Arc, while 81.7% were rhythmic in the dorsal medial hypothalamus. Furthermore, the oscillations within the dorsal Arc had a greater amplitude than within the ventral Arc. This demonstrates that within individual brain regions, unique intrinsic properties leading to nuanced circadian gene expression may exist, and the contribution of distinct neuronal subtypes to this heterogeneity still needs to be determined.

In most of the brain areas we studied, BMAL1 expression was higher than PER2. It is possible that this can be attributed to differences in the efficiency of the antibodies used. Both antibodies have been verified and used extensively in our lab and by others BMAL1 [6, 18, 36, 62, 63] and so we are confident in their specificity. Nonetheless the PER2 antibody is a more difficult antibody to work with and produces higher background, so it is possible that some cells were missed while counting. It is also possible that PER2 is expressed in a more limited number of neurons than BMAL1. In the SCN, Riddle et al. [64] have found that PER1 and PER2 show regional differences in the intensity of their expression and that not all cells express both proteins. They also report that only a very small percentage of neurons that express gastrin-releasing peptide, a marker for SCN neurons in the core region, co-express either PER1 or PER2 at ZT12, when peak PER expression occurs as a whole in the SCN. This supports the possibility that PER2 could be expressed in a slightly more distinct set of cells than BMAL1. This phenomenon was most strongly observed in the OB, where we found that PER2 had a much more limited expression pattern as compared to BMAL1. The glomerular layer was the only subregion to express both proteins, while BMAL1 was also expressed extensively throughout the granule layer, PER2 was only additionally expressed at low levels in the mitral cells. A drawback of this study is that BMAL1 and PER2 were not labelled within the same sets of tissue, so we cannot establish if they were expressed in the same cells or not throughout our samples.

Another major question that also remains is how coherent rhythmic clock gene expression is achieved in brain areas receiving a variety of inputs. For example, the dorsal striatum contains two major types of medium spiny neurons, namely neurons that express either the D1- or the D2-class dopamine receptors. Dopamine has opposing effects on these neurons through G protein-coupled signaling that activates the PKA pathway in D1-receptor bearing neurons or inhibits it in neurons with D2-receptors [65]. Hood et al [20] demonstrated that dopamine was important for producing clock gene rhythms in the dorsal striatum, specifically implicated D2 receptor activity in PER2 entrainment. The results shown here indicate that differences in the ability of D1- and D2-receptors to regulate clock gene expression are not due to differences in these cells’ ability to express clock genes. If PER2 rhythms exist is in both cell types, then, are both cell types entrained to the same signal? Hood et al. [20] may not have examined the correct conditions to properly implicate D1-receptors in clock gene entrainment in the striatum. This is supported by a recent study by Gallardo et al. [66] who showed that D1-receptor knock-out impairs *Per2* rhythms in the dorsal striatum, and daily injections of a D1-receptor agonist could cause behavioural entrainment in intact rats. This indicates that like D2-receptors, D1-receptors can contribute to aspects of circadian control. However, this does not explain how two opposite signaling pathways on separate sets of neurons can lead to cohesive rhythms within the same tissue.

The OB has its own local circuitry and a variety of neuronal cell types, making it an interesting brain area to study in uncovering the mechanisms of circadian gene expression within non-SCN brain regions. Unlike most structures studied here, The OB was unique in that it only expressed PER2 and BMAL1 in a minority of the cells counted (PER2 18.8%, BMAL1 28.0%), and were not found in all layers. Similar results have been reported elsewhere [67, 68]. Interestingly, this structure is one of the few, including the SCN, that can generate autonomous clock gene rhythms [48], yet much is still to be understood about regulation of clock gene expression here. The SCN entrains primarily to light, but the main zeitgeber (environmental entrainment cue) in the OB is not yet understood. It receives phase information from the SCN, yet it will continue to oscillate without SCN input, in behaviourally arrhythmic animals and in isolated tissue cultures [48, 68]. Circadian output from the mitral cells appears to influence olfactory responsivity [69, 70], but despite extensive connectivity to limbic centres and a potential role in complex behaviours in rodents [71], no one has yet been able to link the removal of the OB to circadian behaviours such as locomotor activity patterns or food anticipatory effects [67, 72].

Understanding how circadian gene expression is controlled throughout the brain will be important for understanding how circadian processes contribute to behavior and disease development. The findings made here, that many brain areas express clock proteins across a variety of neural cell types, open the door to more questions. For example, do these different cell types contribute to variations in phase, amplitude or rhythmicity of circadian protein oscillations, or do different proportions of neurons express each of the clock genes? It is likely that many of the properties that exist in the SCN can be applied throughout the brain. However, since each brain region has its own unique inputs, circuitry and neural phenotypes, it is likely that each brain region will also have its own unique intrinsic signaling properties that allow it to produce cohesive circadian gene expression patterns at a tissue level.

## References

[1] Sukumaran S, Almon RR, DuBois DC, Jusko WJ. Circadian rhythms in gene expression: Relationship to physiology, disease, drug disposition and drug action. Adv Drug Deliv Rev. 2010;62(9–10):904–17. 10.1016/j.addr.2010.05.009 20542067 PMC2922481

[2] Videnovic A, Lazar AS, Barker RA, Overeem S. 'The clocks that time us'—circadian rhythms in neurodegenerative disorders. Nature reviews Neurology. 2014;10(12):683–93. Epub 2014/11/12. 10.1038/nrneurol.2014.206 25385339 PMC4344830

[3] Silver R, Kriegsfeld LJ. Circadian rhythms have broad implications for understanding brain and behavior. Eur J Neurosci. 2014;39(11):1866–80. 10.1111/ejn.12593 24799154 PMC4385795

[4] Colwell CS. Linking neural activity and molecular oscillations in the SCN. Nat Rev Neurosci. 2011;12(10):553–69. Epub 2011/09/03. 10.1038/nrn3086 21886186 PMC4356239

[5] Dibner C. On the robustness of mammalian circadian oscillators. Cell cycle. 2009;8(5):681–2. 19223765

[6] Harbour VL, Weigl Y, Robinson B, Amir S. Comprehensive mapping of regional expression of the clock protein PERIOD2 in rat forebrain across the 24-h day. PLoS One. 2013;8(10):e76391 Epub 2013/10/15. 10.1371/journal.pone.0076391 24124556 PMC3790676

[7] Namihira M, Honma S, Abe H, Tanahashi Y, Ikeda M, Honma K. Daily variation and light responsiveness of mammalian clock gene, Clock and BMAL1, transcripts in the pineal body and different areas of brain in rats. Neurosci Lett. 1999;267(1):69–72. Epub 1999/07/10. 10400251 10.1016/s0304-3940(99)00324-9

[8] Masubuchi S, Honma S, Abe H, Ishizaki K, Namihira M, Ikeda M, et al Clock genes outside the suprachiasmatic nucleus involved in manifestation of locomotor activity rhythm in rats. Eur J Neurosci. 2000;12(12):4206–14. 11122332

[9] Shieh KR. Distribution of the rhythm-related genes rPERIOD1, rPERIOD2, and rCLOCK, in the rat brain. Neuroscience. 2003;118(3):831–43. Epub 2003/04/25. 12710990 10.1016/s0306-4522(03)00004-6

[10] Asai M, Yoshinobu Y, Kaneko S, Mori A, Nikaido T, Moriya T, et al Circadian profile of Per gene mRNA expression in the suprachiasmatic nucleus, paraventricular nucleus, and pineal body of aged rats. J Neurosci Res. 2001;66(6):1133–9. 10.1002/jnr.10010 11746446

[11] Smarr BL, Jennings KJ, Driscoll JR, Kriegsfeld LJ. A time to remember: the role of circadian clocks in learning and memory. Behav Neurosci. 2014;128(3):283–303. 10.1037/a0035963 24708297 PMC4385793

[12] Kafka MS, Benedito MA, Blendy JA, Tokola NS. Circadian rhythms in neurotransmitter receptors in discrete rat brain regions. Chronobiology international. 1986;3(2):91–100. 2824075 10.3109/07420528609066353

[13] Kafka MS, Benedito MA, Roth RH, Steele LK, Wolfe WW, Catravas GN. Circadian rhythms in catecholamine metabolites and cyclic nucleotide production. Chronobiology international. 1986;3(2):101–15. 2824067 10.3109/07420528609066354

[14] Castaneda TR, de Prado BM, Prieto D, Mora F. Circadian rhythms of dopamine, glutamate and GABA in the striatum and nucleus accumbens of the awake rat: modulation by light. Journal of pineal research. 2004;36(3):177–85. Epub 2004/03/11. 15009508 10.1046/j.1600-079x.2003.00114.x

[15] Amir S, Robinson B. Thyroidectomy alters the daily pattern of expression of the clock protein, PER2, in the oval nucleus of the bed nucleus of the stria terminalis and central nucleus of the amygdala in rats. Neurosci Lett. 2006;407(3):254–7. Epub 2006/09/16. 10.1016/j.neulet.2006.08.057 16973268

[16] Amir S, Lamont EW, Robinson B, Stewart J. A circadian rhythm in the expression of PERIOD2 protein reveals a novel SCN-controlled oscillator in the oval nucleus of the bed nucleus of the stria terminalis. J Neurosci. 2004;24(4):781–90. Epub 2004/01/30. 10.1523/JNEUROSCI.4488-03.2004 14749422 PMC6729822

[17] Lamont EW, Robinson B, Stewart J, Amir S. The central and basolateral nuclei of the amygdala exhibit opposite diurnal rhythms of expression of the clock protein Period2. Proc Natl Acad Sci U S A. 2005;102(11):4180–4. Epub 2005/03/05. 10.1073/pnas.0500901102 15746242 PMC554834

[18] Segall LA, Amir S. Exogenous corticosterone induces the expression of the clock protein, PERIOD2, in the oval nucleus of the bed nucleus of the stria terminalis and the central nucleus of the amygdala of adrenalectomized and intact rats. J Mol Neurosci. 2010;42(2):176–82. Epub 2010/04/28. 10.1007/s12031-010-9375-4 20422314

[19] Segall LA, Perrin JS, Walker CD, Stewart J, Amir S. Glucocorticoid rhythms control the rhythm of expression of the clock protein, Period2, in oval nucleus of the bed nucleus of the stria terminalis and central nucleus of the amygdala in rats. Neuroscience. 2006;140(3):753–7. 10.1016/j.neuroscience.2006.03.037. 10.1016/j.neuroscience.2006.03.037 16678973

[20] Hood S, Cassidy P, Cossette MP, Weigl Y, Verwey M, Robinson B, et al Endogenous dopamine regulates the rhythm of expression of the clock protein PER2 in the rat dorsal striatum via daily activation of D2 dopamine receptors. J Neurosci. 2010;30(42):14046–58. Epub 2010/10/22. 10.1523/JNEUROSCI.2128-10.2010 20962226 PMC6634752

[21] Gravotta L, Gavrila AM, Hood S, Amir S. Global depletion of dopamine using intracerebroventricular 6-hydroxydopamine injection disrupts normal circadian wheel-running patterns and PERIOD2 expression in the rat forebrain. J Mol Neurosci. 2011;45(2):162–71. Epub 2011/04/13. 10.1007/s12031-011-9520-8 21484443

[22] Gerfen CR, Engber TM, Mahan LC, Susel Z, Chase TN, Monsma FJ Jr. et al D1 and D2 dopamine receptor-regulated gene expression of striatonigral and striatopallidal neurons. Science. 1990;250(4986):1429–32. Epub 1990/12/07. 2147780 10.1126/science.2147780

[23] Lu XY, Ghasemzadeh MB, Kalivas PW. Expression of D1 receptor, D2 receptor, substance P and enkephalin messenger RNAs in the neurons projecting from the nucleus accumbens. Neuroscience. 1998;82(3):767–80. Epub 1998/03/04. 9483534 10.1016/s0306-4522(97)00327-8

[24] Day HE, Curran EJ, Watson SJ Jr, Akil H. Distinct neurochemical populations in the rat central nucleus of the amygdala and bed nucleus of the stria terminalis: evidence for their selective activation by interleukin-1beta. The Journal of comparative neurology. 1999;413(1):113–28. Epub 1999/08/28. 10464374

[25] Swanson LW, Sawchenko PE. Hypothalamic integration: organization of the paraventricular and supraoptic nuclei. Annual review of neuroscience. 1983;6:269–324. Epub 1983/01/01. 10.1146/annurev.ne.06.030183.001413 6132586

[26] Ceccatelli S, Eriksson M, Hökfelt T. Distribution and Coexistence of Corticotropin-Releasing Factor-, Neurotensin-, Enkephalin-, Cholecystokinin-, Galanin- and Vasoactive Intestinal Polypeptide/Peptide Histidine Isoleucine-Like Peptides in the Parvocellular Part of the Paraventricular Nucleus. Neuroendocrinology. 1989;49(3):309–23. 2469987 10.1159/000125133

[27] Vanderhaeghen JJ, Lotstra F, Liston DR, Rossier J. Proenkephalin, [Met]enkephalin, and oxytocin immunoreactivities are colocalized in bovine hypothalamic magnocellular neurons. Proceedings of the National Academy of Sciences. 1983;80(16):5139–43. 10.1073/pnas.80.16.5139 PMC384205 6576380

[28] Yamada K, Emson P, Hokfelt T. Immunohistochemical mapping of nitric oxide synthase in the rat hypothalamus and colocalization with neuropeptides. Journal of chemical neuroanatomy. 1996;10(3–4):295–316. Epub 1996/06/01. 8811420 10.1016/0891-0618(96)00133-0

[29] Olster DH, Blaustein JD. Immunocytochemical colocalization of progestin receptors and beta-endorphin or enkephalin in the hypothalamus of female guinea pigs. Journal of neurobiology. 1990;21(5):768–80. Epub 1990/07/01. 10.1002/neu.480210510 2144316

[30] Foo KS, Hellysaz A, Broberger C. Expression and colocalization patterns of calbindin-D28k, calretinin and parvalbumin in the rat hypothalamic arcuate nucleus. Journal of chemical neuroanatomy. 2014;61–62:20–32. Epub 2014/07/12. 10.1016/j.jchemneu.2014.06.008 25014433

[31] Everitt BJ, Meister B, Hokfelt T, Melander T, Terenius L, Rokaeus A, et al The hypothalamic arcuate nucleus-median eminence complex: immunohistochemistry of transmitters, peptides and DARPP-32 with special reference to coexistence in dopamine neurons. Brain research. 1986;396(2):97–155. Epub 1986/06/01. 2874874 10.1016/s0006-8993(86)80192-5

[32] Lecourtier L, Kelly PH. A conductor hidden in the orchestra? Role of the habenular complex in monoamine transmission and cognition. Neuroscience and biobehavioral reviews. 2007;31(5):658–72. Epub 2007/03/24. 10.1016/j.neubiorev.2007.01.004 17379307

[33] Khachaturian H, Lewis ME, Hollt V, Watson SJ. Telencephalic enkephalinergic systems in the rat brain. J Neurosci. 1983;3(4):844–55. 6834107 10.1523/JNEUROSCI.03-04-00844.1983 PMC6564446

[34] Paxinos G, Watson C. The rat brain in stereotaxic coordinates. 4th ed SanDiego, CA: Academic Press; 1998.

[35] Watson RE Jr, Wiegand SJ, Clough RW, Hoffman GE. Use of cryoprotectant to maintain long-term peptide immunoreactivity and tissue morphology. Peptides. 1986;7(1):155–9. 3520509 10.1016/0196-9781(86)90076-8

[36] Lee IT, Chang AS, Manandhar M, Shan Y, Fan J, Izumo M, et al Neuromedin s-producing neurons act as essential pacemakers in the suprachiasmatic nucleus to couple clock neurons and dictate circadian rhythms. Neuron. 2015;85(5):1086–102. Epub 2015/03/06. 10.1016/j.neuron.2015.02.006 25741729 PMC5811223

[37] Schnell SA, Staines WA, Wessendorf MW. Reduction of lipofuscin-like autofluorescence in fluorescently labeled tissue. J Histochem Cytochem. 1999;47(6):719–30. 10.1177/002215549904700601 10330448

[38] Swanson LW. Brain maps: structure of the rat brain: a laboratory guide with printed and electronic templates for data, models, and schematics. Amsterdam; New York: Elsevier; 2004.

[39] Gangarossa G, Espallergues J, de Kerchove d'Exaerde A, El Mestikawy S, Gerfen CR, Herve D, et al Distribution and compartmental organization of GABAergic medium-sized spiny neurons in the mouse nucleus accumbens. Frontiers in neural circuits. 2013;7:22 Epub 2013/02/21. 10.3389/fncir.2013.00022 23423476 PMC3575607

[40] Ikemoto S. Dopamine reward circuitry: two projection systems from the ventral midbrain to the nucleus accumbens-olfactory tubercle complex. Brain research reviews. 2007;56(1):27–78. Epub 2007/06/19. 10.1016/j.brainresrev.2007.05.004 17574681 PMC2134972

[41] Alheid GF. Extended amygdala and basal forebrain. Annals of the New York Academy of Sciences. 2003;985:185–205. Epub 2003/05/02. 12724159 10.1111/j.1749-6632.2003.tb07082.x

[42] Welsh DK, Takahashi JS, Kay SA. Suprachiasmatic nucleus: cell autonomy and network properties. Annual review of physiology. 2010;72:551–77. Epub 2010/02/13. 10.1146/annurev-physiol-021909-135919 20148688 PMC3758475

[43] Ferguson AV, Latchford KJ, Samson WK. The paraventricular nucleus of the hypothalamus—a potential target for integrative treatment of autonomic dysfunction. Expert opinion on therapeutic targets. 2008;12(6):717–27. Epub 2008/05/16. 10.1517/14728222.12.6.717 18479218 PMC2682920

[44] Joly-Amado A, Cansell C, Denis RG, Delbes AS, Castel J, Martinez S, et al The hypothalamic arcuate nucleus and the control of peripheral substrates. Best practice & research Clinical endocrinology & metabolism. 2014;28(5):725–37. Epub 2014/09/27. 25256767 10.1016/j.beem.2014.03.003

[45] Chronwall BM. Anatomy and physiology of the neuroendocrine arcuate nucleus. Peptides. 1985;6 Suppl 2:1–11. Epub 1985/01/01. 10.1016/0196-9781(85)90128-7 2417205

[46] Warden MK, Young WS. Distribution of cells containing mRNAs encoding substance P and neurokinin B in the rat central nervous system. The Journal of comparative neurology. 1988;272(1):90–113. 10.1002/cne.902720107 2454979

[47] Viswanath H, Carter AQ, Baldwin PR, Molfese DL, Salas R. The medial habenula: still neglected. Frontiers in human neuroscience. 2013;7:931 Epub 2014/01/31. 10.3389/fnhum.2013.00931 24478666 PMC3894476

[48] Abe M, Herzog ED, Yamazaki S, Straume M, Tei H, Sakaki Y, et al Circadian rhythms in isolated brain regions. J Neurosci. 2002;22(1):350–6. Epub 2002/01/05. 11756518 10.1523/JNEUROSCI.22-01-00350.2002 PMC6757616

[49] Albanese A, Altavista MC, Rossi P. Organization of central nervous system dopaminergic pathways. J Neural Transm Suppl. 1986;22:3–17. 3465873

[50] Coronas V, Srivastava LK, Liang JJ, Jourdan F, Moyse E. Identification and localization of dopamine receptor subtypes in rat olfactory mucosa and bulb: a combined in situ hybridization and ligand binding radioautographic approach. Journal of chemical neuroanatomy. 1997;12(4):243–57. Epub 1997/05/01. 9243344 10.1016/s0891-0618(97)00215-9

[51] Levey AI, Hersch SM, Rye DB, Sunahara RK, Niznik HB, Kitt CA, et al Localization of D1 and D2 dopamine receptors in brain with subtype-specific antibodies. Proc Natl Acad Sci U S A. 1993;90(19):8861–5. Epub 1993/10/01. 8415621 10.1073/pnas.90.19.8861 PMC47460

[52] Gerfen CR, Young WS 3rd. Distribution of striatonigral and striatopallidal peptidergic neurons in both patch and matrix compartments: an in situ hybridization histochemistry and fluorescent retrograde tracing study. Brain research. 1988;460(1):161–7. Epub 1988/09/13. 2464402 10.1016/0006-8993(88)91217-6

[53] Gangarossa G, Espallergues J, Mailly P, De Bundel D, de Kerchove d'Exaerde A, Herve D, et al Spatial distribution of D1R- and D2R-expressing medium-sized spiny neurons differs along the rostro-caudal axis of the mouse dorsal striatum. Frontiers in neural circuits. 2013;7:124 Epub 2013/08/03. 10.3389/fncir.2013.00124 23908605 PMC3725430

[54] Skoufias DA, Wilson L. Mechanism of inhibition of microtubule polymerization by colchicine: inhibitory potencies of unliganded colchicine and tubulin-colchicine complexes. Biochemistry. 1992;31(3):738–46. 1731931 10.1021/bi00118a015

[55] Liu B, Kwok RPS, Fernstrom JD. Colchicine-induced increases in immunoreactive neuropeptide levels in hypothalamus: Use as an index of biosynthesis. Life Sciences. 1991;49(5):345–52. 10.1016/0024-3205(91)90441-D. 1677440

[56] Maywood ES, Reddy AB, Wong GK, O'Neill JS, O'Brien JA, McMahon DG, et al Synchronization and maintenance of timekeeping in suprachiasmatic circadian clock cells by neuropeptidergic signaling. Current biology: CB. 2006;16(6):599–605. Epub 2006/03/21. 10.1016/j.cub.2006.02.023 16546085

[57] Choi S, Wong LS, Yamat C, Dallman MF. Hypothalamic ventromedial nuclei amplify circadian rhythms: do they contain a food-entrained endogenous oscillator? J Neurosci. 1998;18. 10.1523/JNEUROSCI.18-10-03843.1998 PMC6793147 9570813

[58] Harbour V. Comprehensive mapping of PERIOD2 expression patterns in the rat forebrain across the 24-hr day. Montreal: Concordia University; 2011.

[59] Reppert SM, Weaver DR. Molecular analysis of mammalian circadian rhythms. Annual review of physiology. 2001;63:647–76. Epub 2001/02/22. 10.1146/annurev.physiol.63.1.647 11181971

[60] Yan L, Karatsoreos I, Lesauter J, Welsh DK, Kay S, Foley D, et al Exploring spatiotemporal organization of SCN circuits. Cold Spring Harbor symposia on quantitative biology. 2007;72:527–41. Epub 2008/04/19. 10.1101/sqb.2007.72.037 18419312 PMC3281753

[61] Guilding C, Hughes AT, Brown TM, Namvar S, Piggins HD. A riot of rhythms: neuronal and glial circadian oscillators in the mediobasal hypothalamus. Molecular Brain. 2009;2(1):28. 19712475 10.1186/1756-6606-2-28 PMC2745382

[62] Uchida H, Nakamura TJ, Takasu NN, Todo T, Sakai T, Nakamura W. Cryptochrome-dependent circadian periods in the arcuate nucleus. Neuroscience Letters. 2016;610:123–8. 10.1016/j.neulet.2015.10.071. 10.1016/j.neulet.2015.10.071 26542738

[63] Delezie J, Dumont S, Sandu C, Reibel S, Pevet P, Challet E. Rev-erbalpha in the brain is essential for circadian food entrainment. Scientific reports. 2016;6:29386 Epub 2016/07/07. 10.1038/srep29386 27380954 PMC4933951

[64] Riddle M, Mezias E, Foley D, LeSauter J, Silver R. Differential localization of PER1 and PER2 in the brain master circadian clock. Eur J Neurosci. 2016. Epub 2016/10/16. 10.1111/ejn.13441 PMC5392174 27740710

[65] Tritsch NX, Sabatini BL. Dopaminergic modulation of synaptic transmission in cortex and striatum. Neuron. 2012;76(1):33–50. Epub 2012/10/09. 10.1016/j.neuron.2012.09.023 23040805 PMC4386589

[66] Gallardo CM, Darvas M, Oviatt M, Chang CH, Michalik M, Huddy TF, et al Dopamine receptor 1 neurons in the dorsal striatum regulate food anticipatory circadian activity rhythms in mice. eLife. 2014;3:e03781 Epub 2014/09/14. 10.7554/eLife.03781 25217530 PMC4196120

[67] Granados-Fuentes D, Saxena MT, Prolo LM, Aton SJ, Herzog ED. Olfactory bulb neurons express functional, entrainable circadian rhythms. Eur J Neurosci. 2004;19(4):898–906. Epub 2004/03/11. 15009137 10.1111/j.0953-816x.2004.03117.x PMC3474850

[68] Granados-Fuentes D, Prolo LM, Abraham U, Herzog ED. The suprachiasmatic nucleus entrains, but does not sustain, circadian rhythmicity in the olfactory bulb. J Neurosci. 2004;24(3):615–9. Epub 2004/01/23. 10.1523/JNEUROSCI.4002-03.2004 14736846 PMC6729269

[69] Granados-Fuentes D, Tseng A, Herzog ED. A circadian clock in the olfactory bulb controls olfactory responsivity. J Neurosci. 2006;26(47):12219–25. Epub 2006/11/24. 10.1523/JNEUROSCI.3445-06.2006 17122046 PMC6675419

[70] Amir S, Cain S, Sullivan J, Robinson B, Stewart J. In rats, odor-induced Fos in the olfactory pathways depends on the phase of the circadian clock. Neurosci Lett. 1999;272(3):175–8. Epub 1999/10/03. 10505609 10.1016/s0304-3940(99)00609-6

[71] Slotnick B. Animal cognition and the rat olfactory system. Trends Cogn Sci. 2001;5(5):216–22. Epub 2001/04/27. 11323267 10.1016/s1364-6613(00)01625-9

[72] Davidson AJ, Aragona BJ, Werner RM, Schroeder E, Smith JC, Stephan FK. Food-anticipatory activity persists after olfactory bulb ablation in the rat. Physiol Behav. 2001;72(1–2):231–5. Epub 2001/03/10. 11240001 10.1016/s0031-9384(00)00417-0

